# Recombination in *Streptococcus pneumoniae* Lineages Increase with Carriage Duration and Size of the Polysaccharide Capsule

**DOI:** 10.1128/mBio.01053-16

**Published:** 2016-09-27

**Authors:** Chrispin Chaguza, Cheryl P. Andam, Simon R. Harris, Jennifer E. Cornick, Marie Yang, Laura Bricio-Moreno, Arox W. Kamng’ona, Julian Parkhill, Neil French, Robert S. Heyderman, Aras Kadioglu, Dean B. Everett, Stephen D. Bentley, William P. Hanage

**Affiliations:** a Department of Clinical Infection, Microbiology and Immunology, Institute of Infection and Global Health, University of Liverpool, Liverpool, United Kingdom; b Microbial Ecology, Malawi-Liverpool-Wellcome Trust Clinical Research Programme, University of Malawi, College of Medicine, Blantyre, Malawi; c Department of Epidemiology, Harvard T.H. Chan School of Public Health, Boston, Massachusetts, USA; d Pathogen Genomics, Wellcome Trust Sanger Institute, Wellcome Trust Genome Campus, Hinxton, Cambridge, United Kingdom; e Department of Biomedical Sciences, University of Malawi, College of Medicine, Blantyre, Malawi; f Division of Infection and Immunity, University College London, London, United Kingdom

## Abstract

*Streptococcus pneumoniae* causes a high burden of invasive pneumococcal disease (IPD) globally, especially in children from resource-poor settings. Like many bacteria, the pneumococcus can import DNA from other strains or even species by transformation and homologous recombination, which has allowed the pneumococcus to evade clinical interventions such as antibiotics and pneumococcal conjugate vaccines (PCVs). Pneumococci are enclosed in a complex polysaccharide capsule that determines the serotype; the capsule varies in size and is associated with properties including carriage prevalence and virulence. We determined and quantified the association between capsule and recombination events using genomic data from a diverse collection of serotypes sampled in Malawi. We determined both the amount of variation introduced by recombination relative to mutation (the relative rate) and how many individual recombination events occur per isolate (the frequency). Using univariate analyses, we found an association between both recombination measures and multiple factors associated with the capsule, including duration and prevalence of carriage. Because many capsular factors are correlated, we used multivariate analysis to correct for collinearity. Capsule size and carriage duration remained positively associated with recombination, although with a reduced *P* value, and this effect may be mediated through some unassayed additional property associated with larger capsules. This work describes an important impact of serotype on recombination that has been previously overlooked. While the details of how this effect is achieved remain to be determined, it may have important consequences for the serotype-specific response to vaccines and other interventions.

## IMPORTANCE

The capsule determines >90 different pneumococcal serotypes, which vary in capsule size, virulence, duration, and prevalence of carriage. Current serotype-specific vaccines elicit anticapsule antibodies. Pneumococcus can take up exogenous DNA by transformation and insert it into its chromosome by homologous recombination. This mechanism has disseminated drug resistance and generated vaccine escape variants. It is hence crucial to pneumococcal evolutionary response to interventions, but there has been no systematic study quantifying whether serotypes vary in recombination and whether this is associated with serotype-specific properties such as capsule size or carriage duration. Larger capsules could physically inhibit DNA uptake, or given the longer carriage duration for larger capsules, this may promote recombination. We find that recombination varies among capsules and is associated with capsule size, carriage duration, and carriage prevalence and negatively associated with invasiveness. The consequence of this work is that serotypes with different capsules may respond differently to selective pressures like vaccines.

## INTRODUCTION

The global mortality due to invasive pneumococcal disease (IPD) has been estimated at more than 1 million per annum, with the majority of deaths occurring in children less than 5 years old (1). Use of a 7-valent pneumococcal conjugate vaccine (PCV7) and subsequent 13-valent (PCV13) formulations has been followed by a precipitate reduction in IPD due to vaccine serotypes (VTs) in the United States, including a substantial herd effect in unvaccinated individuals (2, 3). PCVs are plainly an effective selective pressure on the pneumococcal population. The response to that pressure has been notable for the increase in prevalence of nonvaccine serotypes (NVTs). More than 90 distinct serotypes have been described (4−10), so even PCV13 protects against a small minority of antigenic diversity in this organism. Following vaccination, the prevalence of NVTs in carriage, and to a lesser extent in IPD, has increased as the NVTs took advantage of the removal of their competitors in a process termed serotype replacement (11−13). An important component of serotype replacement has been the success of “capsule switch” vaccine escape variants (14). These variants are lineages that were previously associated with a vaccine serotype but have persisted in the postvaccine environment by the introduction of genes for an NVT capsule through homologous recombination (15). The known ability of the pneumococcus to undergo homologous recombination has also been associated with the acquisition of drug resistance (16, 17) and numerous examples of capsule switch variants (15, 18−20). As a result of this recombination-mediated shuffling of serotype and genotype, when PCVs were introduced, the population already contained potential vaccine escape variants, some of which have subsequently become common and important causes of disease.

The polysaccharide capsule is known to influence multiple aspects of pneumococcal biology. Serotypes vary in their nasopharyngeal carriage rates (21), and their propensity to cause IPD was usually expressed as the “invasive potential” in order to account for the various exposures to different serotypes (22−24). Moreover, these properties have been linked to basic biochemical features of the capsule: those capsules with fewer carbons in the repeat unit of the polysaccharide tend to be thicker and exhibit a more negative surface charge (25) and are associated with a longer duration of carriage and a higher carriage prevalence—an outcome that may be linked to enhanced resistance to opsonophagocytic killing (21). Those serotypes more associated with virulence meanwhile, tend to have smaller capsules and shorter durations of carriage (21, 22, 24).

Despite the importance of both recombination and capsule to pneumococcal biology, whether serotypes vary in their recombination rates in nature and what drives it have not been extensively studied. Genomic studies have shown that different pneumococcal lineages plainly vary in the amount that recombination has contributed to their diversification (20, 26) but have not linked this to the different serotypes associated with each lineage. These studies have also suggested that lineages that completely lack capsule may have a higher relative rate of recombination, suggesting that the capsule can impede the uptake of DNA (26). Support for this comes from the observation that mechanisms such as biofilm formation and downregulation of capsule biosynthesis have been reported to facilitate DNA uptake and chromosomal integration *in vitro* (27). This finding suggested that the polysaccharide capsule may inhibit genetic exchange in encapsulated isolates either physically or structurally (28).

There are hence potentially antagonistic roles for serotype in recombination. A larger capsule is associated with a higher prevalence and longer duration of carriage, therefore offering more opportunities to undergo recombination, but conversely, experimental and observational data suggest that the absence of capsule increases transformation and recombination (26, 27). As yet, there has been no attempt to quantify the association between serotype properties, such as capsule size and carriage duration, and the amount of recombination that those serotypes experience. Understanding this is important because as noted, recombination is crucial to the response of pneumococci to medical interventions (19), and if serotypes vary in recombination in a predictable fashion, we might be able to predict those that are a higher risk of developing drug resistance or vaccine escape.

In the present study, we used whole genomes of 439 pneumococcal isolates collected in Malawi, a low-income country in sub-Saharan Africa with high pneumococcal carriage rates (29) and which contains a wide range of serotypes, including serotypes with very low prevalence or that are absent in industrialized countries such as serotype 1 and 5 (18). We quantified the extent to which recombination has contributed to the history of serotype-specific lineages and combined this with data on carriage duration, invasive potential, carriage prevalence, and capsule size to quantitatively investigate the association. Our results demonstrate significant associations between the recombination rate and frequency with the carriage duration, invasive potential, and size of the outer cell wall polysaccharide capsule in pneumococcal isolates.

## RESULTS

We sequenced the genomes of 439 *Streptococcus pneumoniae* clinical isolates collected from the Queen Elizabeth Central Hospital (QECH) in Blantyre, Malawi (southeastern Africa), between 2002 and 2010. The set of samples comprised 364 invasive isolates and 75 carriage isolates and representatives of 48 distinct serotypes. The most common serotypes were serotype 1 with 83 isolates (52 isolates from blood, 28 isolates from cerebral spinal fluid [CSF], and 3 isolates from carriers), serogroup 6 with 46 isolates (25 isolates from blood, 11 isolates from CSF, and 10 isolates from carriers), serotype 5 with 25 isolates (17 isolates from blood, 6 isolates from CSF, and 2 isolates from carriers), and other serotypes (285 isolates; 126 isolates from blood, 99 isolates from CSF, and 60 isolates from carriers) (see Table S1 in the supplemental material). Whole-genome sequencing was done using the HiSeq platform (Illumina, CA, USA) as described in Materials and Methods. The raw sequence reads had an average length of 72.23 bp (95% confidence interval [95% CI], 70.58 to 73.87 bp), average read quality of 33.48 (95% CI, 33.22 to 33.75), and average insert size of 263.30 kb (95% CI, 263.3 to 270.10 kb) per genome (Fig. S1). Consensus *de novo* sequence assemblies were generated (30, 31) with an average of 66.43 contigs (95% CI, 63.65 to 69.22) and an average chromosome size of 2.09 Mb (95% CI, 2.09 to 2.10 Mb) and average contig size of 37.31 kb (95% CI, 35.91 to 38.72 kb) per genome. The phylogeny of all the isolates was constructed using a 0.79-Mb multiple-sequence alignment of 852 concatenated core genes containing 51,389 single nucleotide polymorphisms (SNPs) (32). For the analysis of serotype-specific evolution of lineages identified as described below, SNPs were called against reference genomes (Table S2).

### Population structure.

**FIG 1:**
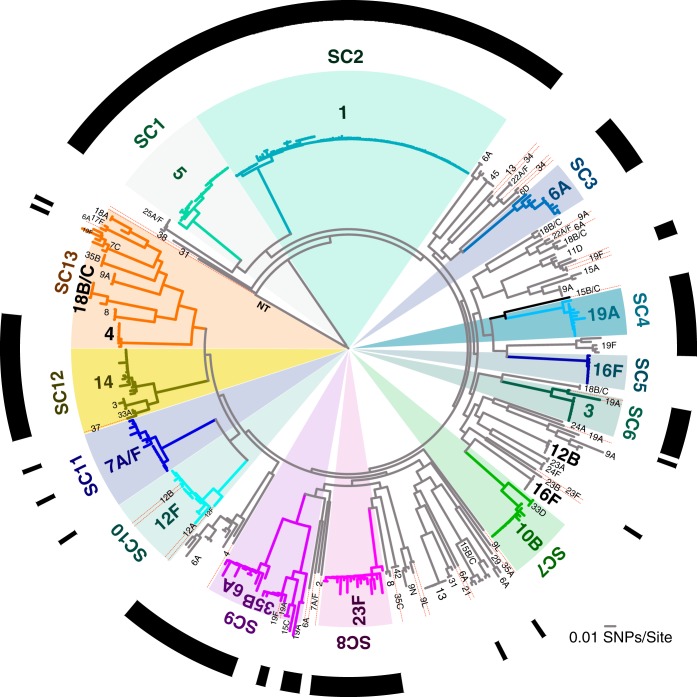
Population structure of *S. pneumoniae* strains from carriers and patients with invasive disease**.** The phylogeny was constructed using a 0.79-Mb multiple-sequence alignment with 51,389 SNPs from 852 universally conserved (core) genes present in single copies. The 14 sequence clusters (SCs) identified by BAPS are labeled on the edge of the phylogeny. Clades corresponding to the 13 different monophyletic SCs (SC1 to SC13) are shown in different colors, while clades for all the strains in the polyphyletic clade (SC14) comprising of the “unclustered” sequences are not colored. The outermost ring around the phylogeny shows whether a serotype is a vaccine type (VT) included in the PCV13 pneumococcal vaccine formulation (black) or nonvaccine type (NVT) not included (white). Phylogeny was rooted using an outgroup method on a branch containing nontypeable (NT) pneumococci, which are genetically distinct from all pneumococcal strains.

Analysis of the population using BAPS (33, 34) identified 14 primary sequence clusters (SCs) (Fig. 1). Of these 14 SCs, SC1 to SC13 were monophyletic clades. The remaining SC, SC14, contained sequences that could not be assigned to a monophyletic SC, and the streptococci in this polyphyletic SC are a diverse group of rare lineages, the grouping of which does not necessarily reflect common evolutionary history, and hence, we undertake no further analysis of this group. Most SCs contained isolates with a single serotype and multiple closely related sequence types (STs) as determined by multilocus sequence typing (MLST) (Fig. 1; see Table S1 in the supplemental material). In some cases, SCs contained multiple serotypes separated by long branches. A notable example is SC9, which consisted of clearly distinct subclades of serotype 6A and 35B (Fig. 1), which we split into two subclades 6A-SC9 and 35B-SC9 for the serotype-specific analyses. The subclade with serotype 35B in SC9 contained additional serotypes with the same ST that were extremely closely related at the whole-genome level (Fig. 1), which suggests that this subclade was a single lineage of serotype 35B that recently acquired other capsule types.

### Variation in recombination among serotypes.

**FIG 2:**
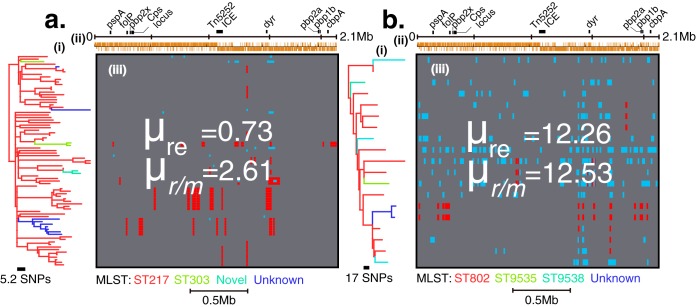
Recombination inferred by Gubbins. Recombination events mapped onto internal nodes (red blocks) and terminal branches (blue blocks) are illustrated for a typical low-recombination serotype (serotype 1 [SC1]) (a) and a high-recombination serotype (serotype 6A [SC3]) (b). (i) Maximum likelihood phylogenetic trees of the different serotypes. Branches and tips of the phylogeny are colored according to the MLST sequence type (ST). (ii) Schematic representation of the reference *S. pneumoniae* genome showing all the genetic annotations and locations of some well-known genes. (iii) Matrix showing the tracks representing each genome. The locations and distribution of regions, which have acquired exogenous DNA through recombination, are colored depending on the number of strains that contain them. Recombination events in internal branches (red) were present in multiple isolates and were shared through clonal descent rather than independent acquisitions, while those in the terminal branches (blue) were isolate specific and represent independent recent acquisitions. The recombination rate (μ_*r*/*m*_), i.e., mean number of the inferred distinct recombination events per isolate (each shared ancestral recombination event that occurred once and spread in the clone via clonal descent was counted once). The recombination frequency (μ_re_), i.e., the mean number of SNPs introduced through recombination to those introduced through mutation, is shown. A high presence of recent rather than shared recombination events implies high μ_re_.

Pneumococci are known to undergo homologous recombination, and all SCs containing capsule switch variants reflect a history of homologous recombination events. To determine the specific recombination events responsible for these changes, as well as those that could have occurred elsewhere in the genome, we used Gubbins (35), which identifies regions with an atypically high density of SNPs using a sliding window approach (Fig. 2a and b; see Fig. S3a to S3o in the supplemental material). We distinguish between two separate recombination parameters. The relative recombination rate (μ_*r*/*m*_) is the ratio of the numbers of SNPs introduced by recombination to the number introduced by mutation. It hence measures the total amount of diversity accumulated by a lineage. In contrast, the recombination frequency (μ_re_) is defined as the average number of recombination events inferred per isolate, regardless of how much variation they have introduced. Hence, a large amount of recombination with closely related strains could lead to a high μ_re_ but a low μ_*r*/*m*_. Furthermore, higher presence of shared ancestral recombination events acquired through clonal descent than strain-specific events introduced independently may lead to a high μ_re_. The averages of both these parameters were calculated for each SC. While SC1 and SC2 both contained isolates with a single serotype (serotype 1 and 5, respectively), in the case of SCs containing more than one serotype, the statistics for each were calculated independently (Table 1). Separate estimates were obtained for the two distinct 6A clades in SC3 and SC9, which we refer to as 6A-SC3 and 6A-SC9, respectively, on the basis of the predominant STs in each.

**TABLE 1:** Summary statistics for the genetic recombination events identified in each serotype

Serotype	Sequence cluster (SC)	No. of isolates (*n*)	Mean no. of recombination events/isolate (μ_re_)	Mean recombination to mutation (μ_*r*/*m*_)		Recombination size (bp)		Pherotype^a^		
				Mean	95% CI^b^	Mean	95% CI	CSP1	CSP2	Other
5	SC1	25	2	2.82	0.29−5.35	7,642	3,877−11,407	100	0	0
1	SC2	83	0.73	2.61	0.74−4.48	8,727	5,753−11,702	98.78	0	1.22
6A	SC3	11	8.91	23.45	1.54−45.44	11,579	8,872−14,286	0	100	0
19A	SC4	12	5.58	11.26	4.64−17.89	9,143	6,308−11,979	0	0	100
16F	SC5	9	0.11	0.44	0−1.39	36,616	0	0	0	100
3	SC6	10	8.1	9.45	0−23.53	8,020	6,201−9,839	0	0	100
10B	SC7	14	2.07	4.15	0−8.70	9,855	4,805−14,905	76.47	17.65	5.88
23F	SC8	19	12.26	12.53	7.33−17.72	8,094	6,737−9,451	94.74	0	5.26
35B	SC9	13	20.08	17.02	5.31−28.74	9,022	7,987−10,056	100	0	0
6A	SC9	12	11.92	12.85	4.55−21.15	7,115	5,697−8,532	100	0	0
12F	SC10	19	5.16	8.17	2.38−13.97	7,709	6,016−9,401	94.74	0	5.26
7A/F	SC11	20	6.4	8.74	3.09−14.38	6,924	5,547−8,302	95	0	5
14	SC12	15	4	13.93	4.70−23.16	6,745	4,926−8,563	100	0	0
18B/C	SC13	8	6.67	3.49	0−11.15	9,469	6,215−12,724	83.78	16.22	0
4	SC13	6	0.75	1.67	0−3.67	9,208	0−20,175	38.75	32.56	28.29

a Proportion of the pneumococcal pherotypes or competence-stimulating peptide (CSP) encoded by the *comC* gene and its variants in each SC. Detailed information about the CSPs is shown in Fig. S2c and S2d in the supplemental material.

b 95% CI, 95% confidence interval.

**FIG 3:**
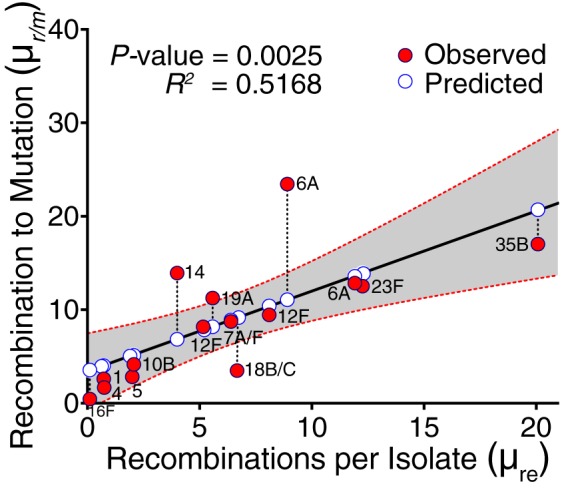
Relationship between rate and frequency of genetic recombination estimates in different *S. pneumoniae* serotypes. The solid red circles show the observed values, while the solid white circles show the estimated values by the univariate linear regression model. The dashed lines connecting the red circles to the white circles represent the residual, the difference between the observed and predicted values by the model. The gray band surrounding the regression line shows the 95% confidence interval (95% CI) for prediction of each data point in the horizontal axis. The two serotype 6A symbols in the figure originated from distinct clades, SC3 and SC9, as shown in Fig. 1 and Table 1.

Estimates of μ_*r*/*m*_ ranged from 0.44 to 23.45 with a mean of 8.89 (95% CI, 5.243 to 12.43) (Table 1), and the only estimate less than 1 was found in the 16F lineage SC5. The highest values of μ_*r*/*m*_ were 12.53 for serotypes 23F-SC8, 23.45 for 6A-SC3, 12.85 for 6A-SC9, and 17.02 for 35B-SC9, while serotypes 1-SC2 (μ_*r*/*m*_ = 2.61), 16F-SC5 (μ_*r*/*m*_ = 0.44), 4-SC13 (μ_*r*/*m*_ = 1.67), and 5-SC1 (μ_*r*/*m*_ = 2.82) showed the lowest values (Table 1). The distribution of μ_*r*/*m*_ values deviated from a normal distribution (*P* < 0.0001) on the basis of three normality tests (see Materials and Methods). In serotypes of the same SC, such as 35B, 6A, and 18B/C in SC4, SC9, and SC13, respectively, the distribution of *r*/*m* ratios per branch were not different with *P* values of 0.7431 and 0.9350, respectively. The average estimated μ_re_ was 6.316 (95% CI, 3.316 to 9.316) and ranged from 0.11 to 20.08. The highest μ_re_ estimates were 12.26 for 23F-SC8, 20.08 for 35B-SC9, 8.91 for 6A-SC3, and 11.92 for 6A-SC9, while serotypes 1-SC2 (μ_re_ = 0.73), 16F-SC5 (μ_re_ = 0.11), 4-SC13 (μ_re_ = 0.75), and 5-SC1 (μ_re_ = 2) showed the lowest μ_re_ values (Table 1). Significant differences (*P* = 0.0005) in the average numbers of recombination events in each serotype were also observed by using the same test (see Fig. S2b in the supplemental material). The Kruskal-Wallis test was used because of significant deviations of the distributions of both μ_*r*/*m*_ per branch in each serotype and the average number of recombination events for each serotype from the normal distribution. Interestingly, both distinct lineages with 6A capsule type consistently showed high μ_*r*/*m*_ (23.45 in SC3 and 12.85 in SC9) and μ_re_ (8.91 in SC3 and 11.92 in SC9) despite having a dissimilar genetic backbone. In addition, μ_*r*/*m*_ positively correlated with μ_re_ (*R^2^* = 0.5168; *P* = 0.0025) (Fig. 3). The pneumococcal pherotypes or competence-stimulating peptides (CSP) encoded by the *comC* gene were diverse and variably distributed in the SCs but showed no obvious association with the recombination parameters (Table 1).

### Serotype-specific carriage duration, prevalence, and invasive potential correlate with recombination.

**FIG 4:**
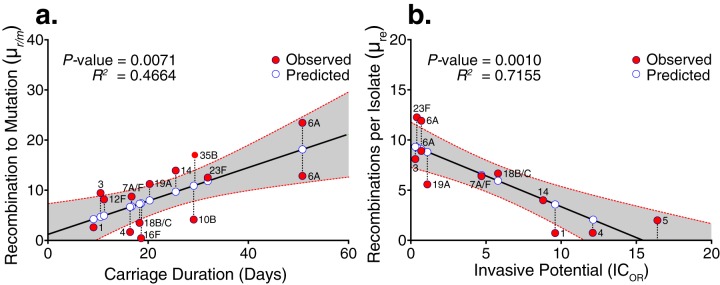
Statistical relationships between recombination rate (μ_*r*/*m*_) and frequency (μ_re_) with carriage duration and serotype invasive potential (IC_OR_). Univariate regression between μ_*r*/*m*_ and carriage duration (a) and μ_re_ and IC_OR_ (invasive to carriage odds ratio) (b). The solid red circles show the observed values, while the solid white circles show the estimated values by the univariate linear regression model. The dashed lines connecting the red circles to the white circles represent the residual, the difference between the observed and predicted values by the model. The gray band surrounding the regression line shows the 95% confidence interval (CI) for prediction of each data point in the horizontal axis. The two serotype 6A symbols in the figure originated from distinct clades, SC3 and SC9, as shown in Fig. 1 and Table 1.

Different pneumococcal lineages vary in the extent of recombination that has influenced their genomes (20, 26). Serotypes are known to vary in their duration of carriage and propensity to cause invasive disease per exposure. A longer duration of carriage could offer more opportunities for cocolonization with different strains and possible recombination. We hence tested the association for each serotype between μ_*r*/*m*_ and μ_re_ and previously published serotype-specific estimates of carriage duration (36), carriage prevalence data from this study, capsule size (21), and invasive potential (defined as the odds ratio for causing invasive disease to carriage [IC_OR_] [22, 37]) using univariate linear regression. We observed a positive association between both μ_*r*/*m*_ (*R^2^* = 0.4664; *P* = 0.0071) and μ_re_ (*R^2^* = 0.3393; *P* = 0.0367) with carriage duration (Fig. 4a and Table 2; see Fig. S4a in the supplemental material), but only μ_*r*/*m*_ showed a significant association (*R^2^* = 0.4173; *P* = 0.0093) with carriage prevalence of the serotypes (Table 2; see Fig. S6a and S6b in the supplemental material). When modeling the relationship between carriage duration and μ_re,_ serotype 35B-SC9 was excluded as an outlier, but its inclusion decreased the *P* value (*R^2^* = 0.2406; *P* = 0.0749). Both μ_*r*/*m*_ (*R^2^* = 0.4549; *P* = 0.0228) and μ_re_ (*R^2^* = 0.7155; *P* = 0.0010) showed negative association with the invasive potential of the studied serotypes (Fig. 4b and Table 2; Fig. S4b and S4c). In multivariable regression, the association of carriage duration, prevalence, and invasive potential disappeared (Table 2). Repeated univariate and multivariate analyses using only those serotypes for which all response variables were available found similar results (Table S3). A multivariate regression to account for this removed the significant association with serotype invasive potential, while the association with carriage duration and capsule size remained (Table 2).

**TABLE 2:** Summarized estimates from the univariate, multivariable, and multivariate multiple regression

Response variable	Predictor variable	Univariate			Multivariable			Multivariate		
		Estimate	SE	*P* value^a^	Estimate	SE	*P* value	Estimate^b^	Approx. *F*	*P* value^a^
Recombination rate (μ_*r*/*m*_)	Capsule size	0.0205	0.0186	0.3000	−0.0185	0.0242	0.487	0.9044	14.183	0.0296*
	Carriage duration	0.3336	0.1030	0.0071*	0.2149	0.3301	0.550	0.9085	14.884	0.0277*
	Invasive potential	−0.7930	0.2894	0.0228*	−0.8847	0.7927	0.327	0.4032	1.0134	0.4611
	Carriage prevalence	1.0030	0.3287	0.0093*	0.1081	0.9468	0.915	0.5457	1.8021	0.3062

Recombination frequency (μ_re_)	Capsule size	0.0263	0.0078	0.0095*	0.0057	0.0081	0.516	0.9044	14.183	0.0296*
	Carriage duration	0.1707	0.0718	0.0367*	0.2595	0.1101	0.078	0.9085	14.884	0.0277*
	Invasive potential	−0.6142	0.1291	0.0010*	−0.4127	0.2645	0.194	0.4032	1.0134	0.4612
	Carriage prevalence	0.5443	0.3260	0.1189	−0.4967	0.3159	0.191	0.5457	1.8021	0.3062

a The asterisks in the *P* value columns indicate significant values.

b All test statistics, i.e., Pillai-Bartlett trace criterion, Wilks’ lambda, Hotelling-Lawley trace, and Roy’s greatest root, were used to summarize the results from the multivariate analysis of variance (MANOVA) of the multivariate multiple regression model. All the tests showed identical *P* values. For illustration, only estimates using the Pillai-Bartlett trace are shown.

### Recombination correlates with larger polysaccharide capsules.

**FIG 5:**
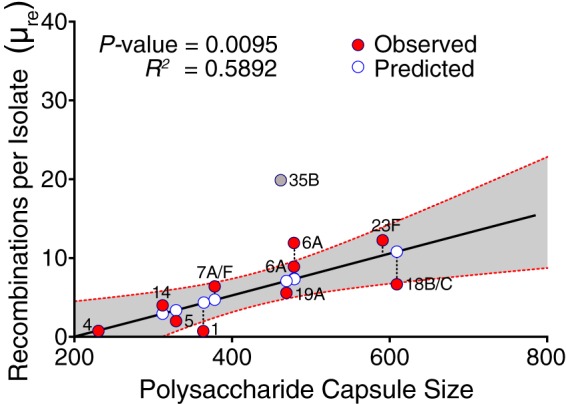
Relationship between frequency of recombination (μ_re_) and polysaccharide capsule size in pneumococcal isolates. Serotype 35-SC9 was excluded from the regression, as it was deemed an outlier observation. The solid red circles show the observed values, while the solid white circles show the estimated values by the univariate linear regression model. The dashed lines connecting the red circles to the white circles representthe residual, the difference between the observed and predicted values by the model. The gray band surrounding the regression line shows the 95% confidence interval (CI) for prediction of each data point in the horizontal axis. The two serotype 6A symbols in the figure originated from distinct clades, SC3 and SC9, as shown in Fig. 1 and Table 1.

Capsule size has been associated with the factors described above—the serotype-specific duration of carriage and prevalence. The polysaccharide capsule size data for different pneumococcal serotypes, determined by the fluorescein isothiocyanate-labeled dextran (FITC-dextran) assay, were obtained from Weinberger and colleagues (21). By using univariate regression, we observed positive association between the capsule size and μ_re_ (*R^2^* = 0.5892; *P* = 0.0095), but not μ_*r*/*m*_ (*R^2^* = 0.1186; *P* = 0.300) (Fig. 5; Table 2). Excluding serotype 35-SC9, which was deemed to be an outlier observation, the linear association between capsule size and μ_*r*/*m*_ increased (*R^2^* = 0.3519; *P* = 0.0544). This was robust to multivariate analysis (*P* = 0.0296). We also did a multivariable regression to account for collinearity among capsule size, duration of carriage, carriage prevalence, and invasive potential and identify the independent contribution of each, which showed no association between μ_*r*/*m*_ and μ_re_ (Table 2; see Table S3 in the supplemental material).

## DISCUSSION

Recombination is a fundamental process in the evolution and adaptation of many pathogens. It is capable of shuffling existing variation into more fit combinations, and in the case of pneumococcus, it has been an important force in its response to medical interventions like vaccines and antibiotics (19). Despite the obvious benefits to the pathogen that recombination offers in this regard, the rates with which it occurs vary greatly among pneumococcal lineages and serotypes (26) for reasons that are obscure. Understanding the factors that contribute to this variation is important as we attempt to limit the potential of pneumococcal evolution to erode the benefits of clinical innovation.

Previous studies have commented on the low presence of recombination in certain serotypes, such as the “highly invasive” serotypes 1 and 7F (20, 38, 39). A systematic comparison of capsular types is challenging because some serotypes are comparatively rare or absent from samples taken in industrialized countries, and while it might be possible to combine studies from different sites, it would be important to account for any bias arising from variation in recombination in different regions—for instance, previous work with MLST data found more evidence of recombination in samples from African carriers and speculated that this might be the consequence of a higher carriage rate (40).

We have used a set of pneumococcal samples that includes an unusually wide range of serotypes with diverse properties, and we found a clear association between serotype and μ_*r*/*m*_ and μ_re_. Because of lack of data in resource-poor settings, we used previously published data on the properties of serotypes, such as capsule size, carriage duration, prevalence, and invasive capacity (all of which vary relatively little among sample sites); we found that all were associated with one recombination parameter or the other recombination parameter or both. In univariate analysis, a negative association with invasive capacity mirrored a positive association of μ_*r*/*m*_ with carriage duration and prevalence—likely reflecting the fact that the majority of highly invasive serotypes are also infrequently carried. However, it should be noted that we did not have specific invasiveness data from pneumococci infecting the Malawi population, and there are few estimates for this quantity from resource-poor settings, so this is an important focus for future work.

We have also found more frequent recombination in serotypes with larger outer cell wall polysaccharide capsules, which shows that despite the potential physical barrier offered by the capsule, the presence of a larger capsule itself does not simply reduce recombination. While this could be considered surprising, it is important to note that serotypes with enlarged capsules are typically carried for longer duration and resist neutrophil phagocytosis (21) and complement-mediated immune responses (41, 42) than serotypes with smaller capsules. In addition, this may be because some of the serotypes with larger capsules have lower metabolic costs (21) associated with capsule expression and may have a higher ability to form biofilms which may prevent elimination (27). This suggests that longer duration of carriage and lower clearance rates by the host’s immune system in serotypes with enlarged capsules leads to sustained exposure to cocolonizers (potential donors of exogenous DNA), which has been previously described to facilitate recombination (40). This in turn results in higher recombination than in serotypes with thinner capsules, which are presented with limited opportunities for recombination due to rapid clearance. The intrinsic recombination rates *in vitro* in the absence of capsule-induced immune clearance may be different than observed in the natural population where various factors such as strain competition and immune responses can directly affect the recombination rate. Hence, the results of *in vitro* studies may be misleading when applied to the nasopharynx. In addition, there was no obvious association between the pneumococcal pherotypes such as CSP1, CSP2, and other variants encoded by the *comC* gene and its allelic variants, which suggests that the observed differences in recombination may not be due to the differences in competence. The evidence presented here certainly strongly suggests that capsule does not prevent recombination in any straightforward fashion. However, while capsule size was associated with a higher frequency of recombination (μ_re_), the association with μ_*r*/*m*_ was not significant in the univariate regression. Nevertheless, the effect of capsule size and carriage duration in a multivariate analysis accounts for the high correlation between the two recombination parameters, i.e., μ_*r*/*m*_ and μ_re_, indicating that these two factors are key contributors to the observed variation. The capsule data used in this study were obtained *in vitro*, and the expressed capsule sizes during carriage and transmission remain unclear and should be investigated in follow-up studies.

The properties of the capsule that we have studied here are also highly correlated (Pearson correlation coefficients are shown in Fig. S7 in the supplemental material), and accounting for this removed the association (Table 2). As a result, we have not been able to precisely define the underlying causes. A plausible mechanism is that serotypes with larger capsules are more exposed to other cocolonizing strains because they are carried for a longer period and at a higher prevalence. One way to test this would be to use serotype switches as natural experiments and compare μ_*r*/*m*_ and μ_re_ between switched lineages where the two serotypes involved vary in their size. However, this is limited by the fact that serotype switches identified in Fig. 1 and other studies typically involve serotypes that are effective colonizers such as serogroups 6, 15, 18, 19, and 23 (15). While this means we cannot use the present data set to do the above analysis, we should note that this observation is consistent with the suggestion that more commonly carried types are more likely to undergo recombination with each other.

In conclusion, we have shown that in a set of samples from Malawi containing many different serotypes, there is a linear relationship between μ_re_ and μ_*r*/*m*_ and properties of the capsule. In general, there is evidence that recombination increases with carriage duration and polysaccharide capsule size. We have not been able to pick out the causal roots of this relationship due to the complexity of the relationships between other important properties also associated with capsule, but the observation shows conclusively that all serotypes are not alike and that serotypes differ in how recombination has affected their evolution history, which has been associated with many different responses from drug resistance to vaccine escape. The serotypes observed in settings with a high burden of carriage and disease, such as sub-Saharan Africa, are often different from those found in industrialized nations, and there has been concern that lineages with serotypes targeted by vaccine such as serotypes 1, 5, and 7F that are relatively invasive, with a short duration of carriage and small capsules, might survive a serotype switch (21, 22, 36). The results of this study suggest that such concerns may be misplaced.

## MATERIALS AND METHODS

### Isolate collection and genome sequencing.

We collected 439 *S. pneumoniae* isolates from the Malawi-Liverpool-Wellcome Trust Clinical Research Programme (MLW) pneumococcal isolate archive in Blantyre, Malawi. The isolates were routinely collected from patients at the Queen Elizabeth Central Hospital, a 1,250-bed referral hospital and the largest hospital located in the Blantyre District in southern Malawi from 2002 to 2011. All isolates were collected before the introduction of the 13-valent pneumococcal conjugate vaccine (PCV13) in routine childhood immunization programs in Malawi in 2011. Isolates were collected from carriers (*n* = 75) and patients with invasive disease (*n* = 364) from 2002 to 2011 (see Table S1 in the supplemental material). Isolates were cultured in Todd-Hewitt broth, and genomic DNA was extracted using the Promega Wizard DNA genomic DNA purification kit (Promega, USA). DNA sequencing was done using the Illumina genome analyzer II (Illumina, CA, USA) platform at the Wellcome Trust Sanger Institute. A robust bespoke pipeline (43) that was composed of the Velvet v1.2.09 De Bruijn graph-based DNA sequence assembler (30), Velvet Optimizer (31), SSPACE Basic v2.0 contig-scaffolding software (44), GapFiller (45), and SMALT v0.7.4 short-read aligner (http://sourceforge.net/projects/smalt/) was used to generate sequence assemblies from the paired-end Illumina sequence reads for the study isolates. Serotypes were identified from the whole-genome sequence data using an *in silico* approach implemented with Perl and BioPerl (46) as described by Croucher et al. (47).

### Population structure and phylogenetic analysis.

Phylogenetic trees were constructed using a 0.79-Mb sequence alignment of 51,389 single nucleotide polymorphisms (SNPs) in 852 concatenated core genes present in single copies (no paralogs). The conserved (core) and nonconserved (accessory) genes were identified using the Roary bacterial genome analysis pipeline (32). The population structure of the isolates was inferred using the hierBAPS module implemented in BAPS v6.0 (33, 34). Maximum likelihood phylogenies of the concatenated core gene alignments for all the study isolates and serotype-specific isolates in the inferred sequence clusters (SCs) from BAPS were constructed using RAxML v7.0.4 (48). We used a generalized time reversible (GTR) (49) model with gamma (Υ) heterogeneity across nucleotide sites and 100 bootstrap replicates. The tree was rooted with an outgroup clade of nontypeable (NT) isolates, which form a distinct clade from other pneumococci (50). Phylogenetic trees and associated metadata, namely, sequence types (STs), serotypes, and inclusion of the sample’s serotype in the PCV13 vaccine formulation, were overlaid on the phylogenies and visualized in iToL (51) and BioPython scripts (52).

### Recombination detection and phylogeny construction.

Phylogenies for specific serotypes were generated for downstream phylogenetic analysis through mapping paired-end short sequence reads against different published reference whole-genome sequences (see Table S2 in the supplemental material) for different pneumococcal serotypes using SMALT v0.7.4. The output binary alignment map (BAM) files were realigned using the Genome Analysis Tool Kit (GATK) v3.3.0 (53). The locations of recombination events in each chromosome in the alignments were detected using Gubbins v1.1.1 (35). The serotype-specific phylogenies with recombination removed were constructed using RAxML v7.0.4 (48) as above and rooted in the middle of the branch, separating the two most divergent isolates. Visualization of the phylogenies was done as described in “Population structure and phylogenetic analysis” above.

### Carriage duration, serotype invasive potential, and capsule size.

We used previously published data from Kilifi in Kenya, data on carriage duration of pneumococcal serotypes in the human nasopharynx from Abdullahi and colleagues (36) and data on serotype invasive potential (odds ratio for IPD to carriage [IC_OR_]) from Brueggemann and colleagues (22). Additional data for serotype 5 invasive potential were obtained from Smith and colleagues (37). The polysaccharide capsule sizes for the serotypes were obtained from Weinberger and colleagues (21) where the degree of encapsulation was determined by measuring the zone of exclusion using the fluorescein isothiocyanate-labeled dextran (FITC-dextran) assay. The specific methods used to obtain the data sets are described in the respective publications. We excluded serotypes with estimated IC_OR_ values of 0 (22) to prevent overfitting as a consequence of the smaller number of isolates used from invasive disease sample collection. Genomic data on the relative recombination rate and frequency of pneumococcal lineages and prevalence of serotypes in carriage were obtained from Malawi (this study).

### Statistical analysis.

We used univariate, multivariable, and multivariate multiple regression to examine the association between known properties of serotypes and the relative recombination rate (μ_*r*/*m*_) and frequency (μ_re_). The univariate regression model were separately formulated as *Y_i_* = β_0_ + β_1_*x_i_* + ε_i_ for each dependent variable *Y_i_*(μ_*r*/*m*_ and μ_re_) against each independent variable *x_i_* where *i* designates the polysaccharide capsule size, carriage duration, serotype invasive potential, and carriage prevalence. The intercept and regression coefficient for the independent variables are represented by β_0_ and β_1_, respectively, while ε_i_ represents the residuals. We formulated the multivariable model similarly but included multiple independent variables to account for collinearity between them as *Y_i_* = β_0_ + β_1_*x_i_* + β_2_*x*_i_ + β_3_*x*_i_ + β_4_*x_i_* + ε_i_, where β_0_ represents the intercept and the terms β_1_, β_2,_ β_3,_ and β_4_ are the regression coefficients for each independent variable (*x_i_*) where *i* is polysaccharide capsule size, carriage duration, serotype invasive potential and carriage prevalence. We also modeled the relationship between both dependent variables (μ_*r*/*m*_ and μ_re_) and the independent variables in the multivariable regression this time with a multivariate multipl e regression model. The multivariate model was formulated as *Y*_n*x*m_ = *X*_n*x*(*p*+1)_β_(*p*+1)*x*m_ + ε_n*x*m_, where *n* designates each serotype and lineage combination as follows 1-SC2, 5-SC1, 6A-SC3,  …, and 4-SC13 (Table 1). The *m* in the matrix *Y*_n*x*m_ takes values 1 and 2 that designate dependent variables μ_*r*/*m*_ and μ_re_, respectively. The model matrix is the term *X*_n*x*(*p*+1)_ and consists of *p*+1 regressors where *p* represents polysaccharide capsule size, carriage duration, serotype invasive potential, and carriage prevalence of serotypes. The first column in the model matrix *X*_n*x*(*p*+1)_ consists of 1*s*, which represents the regression constants. The matrix of regression coefficients for all the independent variables in *X*_n*x*(*p*+1)_ is represented by parameter β. The residual term in the model is represented by the matrix ε_n*x*m_. For comparison with the multivariable and multivariate analyses, we repeated the univariate analyses with the same sample size.

We calculated the pairwise Pearson correlation coefficients for independent and response variables and plotted them using the ggplot2 package. The regression analysis, normality tests, namely, D’Agostino and Pearson, Shapiro-Wilk, and Kolmogorov-Smirnov tests, Kruskal-Wallis and analysis of variance (ANOVA) tests and summary statistics, such as means and ranges, were calculated using R v3.1.2 (R Core Team, 2014) and GraphPad Prism v6.0 (GraphPad Software, Inc., CA, USA).

## SUPPLEMENTAL MATERIAL

Figure S1  Assembly statistics of the pneumococcal study isolates. (a) Length of the assembled genomes; (b) mean number of contigs per genome; (c) mean contig length; (d) *N*_50_ assembly statistic; (e) mean number of contigs in *N*_50_; (f) mean read length; (g) percentage of total mapped sequence reads; (h) percentage of the total bases mapped. Download Figure S1, EPS file, 2.1 MB

Figure S2  Characteristics of the genetic recombination events identified in the pneumococcal isolates. (a) Distribution of recombination rates on branches of the serotype-specific phylogenies; (b) sizes of recombination blocks identified in the genomes of each pneumococcal serotype; (c) distribution of the competence-stimulating peptide (CSP) variants across the sequence clusters (SCs); (d) protein multiple-sequence alignment of the CSPs encoded by the *comC* gene. Other variants of the *comC* gene in additional to the CSP1 and CSP2 were arbitrarily named CPS3 to CSP10. Download Figure S2, TIF file, 1 MB

Figure S3  Recombination inferred by Gubbins. Pneumococcal serotypes 5-SC1 (a), 2-SC1 (b), 6A-SC3 (c), 19A-SC4 (d), 16F-SC5 (e), 3-SC6 (f), 10B-SC7 (g), 23F-SC8 (h), 6A-SC9 (i), 35B-SC9 (j), 12F-SC10 (k), 7AF-SC11 (l), 14-SC12 (m), 4-SC13 (n), and 18 A/F-SC13 (o). (i) Maximum likelihood phylogenetic trees of the different serotypes. Branches and tips of the phylogeny are colored according to the MLST sequence type (ST). (ii) Schematic representation of the reference *S. pneumoniae* genome showing all the genetic annotations and locations of some well-known genes. (iii) Matrix showing the tracks representing each genome. The locations and distribution of regions, which have acquired exogenous DNA through recombination, are colored depending on the number of strains that contain them. Recombination events in internal branches (red) were present in multiple isolates and were shared through clonal descent, rather than independent acquisitions, while those in the terminal branches (blue) were isolate specific and represent independent recent acquisitions. The recombination rate (μ_*r*/*m*_), i.e., mean number of the inferred distinct recombination events per isolate (each shared ancestral recombination event that occurred once and spread in the clone via clonal descent was counted once) and the recombination frequency (μ_re_), i.e., the mean number of SNPs introduced through recombination to those introduced through mutation are shown. A high presence of recent recombination events rather than shared recombination events implies a high μ_re_. Download Figure S3, TIF file, 1.6 MB

Figure S4  Relationships between recombination rate (μ_*r*/*m*_) and frequency (μ_re_) with carriage duration and invasive potential in pneumococcal isolates. Univariate regression between μ_re_ and carriage duration (a) and μ_*r*/*m*_ and IC_OR_ (invasive to carriage odds ratio) (b). The solid red circles show the observed values, while the solid white circles show the estimated values by the univariate linear regression model. The dashed lines connecting the red circles to the white circles represent the residual, the difference between the observed and predicted values by the model. The gray band surrounding the regression line shows the 95% confidence interval (CI) for prediction of each data point in the horizontal axis. The two serotype 6A symbols in the figure originated from distinct clades, SC3 and SC9, as shown in Fig. 1 and Table 1. Download Figure S4, EPS file, 1.9 MB

Figure S5  Recombination dynamics and capsule polysaccharide capsule sizes in pneumococcal serotypes. (a) Recombination frequency (μ_re_) and capsule size. Serotype 35-SC9 (light pink) was excluded, as it was deemed an outlier. (b) Recombination rate (μ_*r*/*m*_) and capsule sizes. The solid red circles show the observed values, while the solid white circles show the estimated values by the univariate linear regression model. The dashed lines connecting the red circles to the white circles represent the residual, the difference between the observed and predicted values by the model. The gray band surrounding the regression line shows the 95% confidence interval (CI) for prediction of each data point in the horizontal axis. The two serotype 6A symbols in the figure originated from distinct clades, SC3 and SC9, as shown in Fig. 1 and Table 1. Download Figure S5, EPS file, 1 MB

Figure S6  Relationship between frequency (μ_re_) of recombination and carriage prevalence in pneumococcal isolates. (a) μ_*r*/*m*_ and carriage prevalence; (b) μ_re_ and carriage prevalence. The solid red circles show the observed values, while the solid white circles show the estimated values by the univariate linear regression model. The dashed lines connecting the red circles to the white circles represent the residual, the difference between the observed and predicted values by the model. The gray band surrounding the regression line shows the 95% confidence interval (CI) for prediction of each data point in the horizontal axis. The two serotype 6A symbols in the figure originated from distinct clades, SC3 and SC9, as shown in Fig. 1 and Table 1. Download Figure S6, EPS file, 1.9 MB

Figure S7  Composite Pearson correlation matrix derived for the variables. The independent (predictor) variables, namely, carriage duration and capsule size, are indicated by two asterisks, while the different dependent (response) variables, namely, recombination rate (μ_*r*/*m*_), recombination frequency (μ_re_), serotype invasive potential (IC_OR_), and prevalence in carriage and invasive disease, do not have an asterisk. Download Figure S7, EPS file, 1.2 MB

Table S1  Streptococcus pneumoniae isolates used in this study. Table S1, DOCX file, 0.2 MB

Table S2  Reference pneumococcal whole-genome sequences used in this study. Table S2, DOCX file, 0.1 MB

Table S3  Summarized estimates from the univariate and multivariable regression. Only serotypes with all the different independent variables present, i.e., capsule size, carriage duration, invasive potential, and carriage prevalence were used. Table S3, DOCX file, 0.1 MB

## References

[1] O’Brien KL, Wolfson LJ, Watt JP, Henkle E, Deloria-Knoll M, McCall N, Lee E, Mulholland K, Levine OS, Cherian T, Hib and Pneumococcal Global Burden of Disease Study Team 2009 Burden of disease caused by Streptococcus pneumoniae in children younger than 5 years: global estimates. Lancet 374:893–902. doi:10.1016/S0140-6736(09)61204-6. 19748398

[2] Moore MR, Link-Gelles R, Schaffner W, Lynfield R, Lexau C, Bennett NM, Petit S, Zansky SM, Harrison LH, Reingold A, Miller L, Scherzinger K, Thomas A, Farley MM, Zell ER, Taylor TH Jr, Pondo T, Rodgers L, McGee L, Beall B, Jorgensen JH, Whitney CG 2015 Effect of use of 13-valent pneumococcal conjugate vaccine in children on invasive pneumococcal disease in children and adults in the USA: analysis of multisite, population-based surveillance. Lancet Infect Dis 15:301–309. doi:10.1016/S1473-3099(14)71081-3. 25656600 PMC4876855

[3] Whitney CG, Farley MM, Hadler J, Harrison LH, Bennett NM, Lynfield R, Reingold A, Cieslak PR, Pilishvili T, Jackson D, Facklam RR, Jorgensen JH, Schuchat A, Active Bacterial Core Surveillance of the Emerging Infections Program Network 2003 Decline in invasive pneumococcal disease after the introduction of protein–polysaccharide conjugate vaccine. N Engl J Med 348:1737–1746. doi:10.1056/NEJMoa022823. 12724479

[4] Bentley SD, Aanensen DM, Mavroidi A, Saunders D, Rabbinowitsch E, Collins M, Donohoe K, Harris D, Murphy L, Quail MA, Samuel G, Skovsted IC, Kaltoft MS, Barrell B, Reeves PR, Parkhill J, Spratt BG 2006 Genetic analysis of the capsular biosynthetic locus from all 90 pneumococcal serotypes. PLoS Genet 2:e31. doi:10.1371/journal.pgen.0020031. PMC1391919 16532061

[5] Calix JJ, Nahm MH 2010 A new pneumococcal serotype, 11E, has a variably inactivated wcjE gene. J Infect Dis 202:29–38. doi:10.1086/653123. 20507232 PMC2880655

[6] Jin P, Kong F, Xiao M, Oftadeh S, Zhou F, Liu C, Russell F, Gilbert GL 2009 First report of putative Streptococcus pneumoniae serotype 6D among nasopharyngeal isolates from Fijian children. J Infect Dis 200:1375–1380. doi:10.1086/606118. 19803727

[7] Park IH, Pritchard DG, Cartee R, Brandao A, Brandileone MC, Nahm MH 2007 Discovery of a new capsular serotype (6C) within serogroup 6 of Streptococcus pneumoniae. J Clin Microbiol 45:1225–1233. doi:10.1128/JCM.02199-06. 17267625 PMC1865839

[8] Oliver MB, van der Linden MP, Küntzel SA, Saad JS, Nahm MH 2013 Discovery of Streptococcus pneumoniae serotype 6 variants with glycosyltransferases synthesizing two differing repeating units. J Biol Chem 288:25976–25985. doi:10.1074/jbc.M113.480152. 23897812 PMC3764802

[9] Calix JJ, Porambo RJ, Brady AM, Larson TR, Yother J, Abeygunwardana C, Nahm MH 2012 Biochemical, genetic, and serological characterization of two capsule subtypes among Streptococcus pneumoniae serotype 20 strains: discovery of a new pneumococcal serotype. J Biol Chem 287:27885–27894. doi:10.1074/jbc.M112.380451. 22736767 PMC3431705

[10] Park IH, Geno KA, Yu J, Oliver MB, Kim KH, Nahm MH 2015 Genetic, biochemical, and serological characterization of a new pneumococcal serotype, 6H, and generation of a pneumococcal strain producing three different capsular repeat units. Clin Vaccine Immunol 22:313–318. doi:10.1128/CVI.00647-14. 25589550 PMC4340893

[11] Hanage WP, Bishop CJ, Huang SS, Stevenson AE, Pelton SI, Lipsitch M, Finkelstein JA 2011 Carried pneumococci in Massachusetts children: the contribution of clonal expansion and serotype switching. Pediatr Infect Dis J 30:302–308. doi:10.1097/INF.0b013e318201a154. 21085049 PMC3175614

[12] Hanage WP, Bishop CJ, Lee GM, Lipsitch M, Stevenson A, Rifas-Shiman SL, Pelton SI, Huang SS, Finkelstein JA 2011 Clonal replacement among 19A Streptococcus pneumoniae in Massachusetts, prior to 13 valent conjugate vaccination. Vaccine 29:8877–8881. doi:10.1016/j.vaccine.2011.09.075. 21964059 PMC3221484

[13] Hanage WP, Finkelstein JA, Huang SS, Pelton SI, Stevenson AE, Kleinman K, Hinrichsen VL, Fraser C 2010 Evidence that pneumococcal serotype replacement in Massachusetts following conjugate vaccination is now complete. Epidemics 2:80–84. doi:10.1016/j.epidem.2010.03.005. 21031138 PMC2963072

[14] Brueggemann AB, Pai R, Crook DW, Beall B 2007 Vaccine escape recombinants emerge after pneumococcal vaccination in the United States. PLoS Pathog 3:e168. 10.1371/journal.ppat.0030168 PMC2077903 18020702

[15] Croucher NJ, Kagedan L, Thompson CM, Parkhill J, Bentley SD, Finkelstein JA, Lipsitch M, Hanage WP 2015 Selective and genetic constraints on pneumococcal serotype switching. PLoS Genet 11:e01053-16. doi:10.1371/journal.pgen.1005095. PMC4380333 25826208

[16] Hanage WP, Fraser C, Tang J, Connor TR, Corander J 2009 Hyper-recombination, diversity, and antibiotic resistance in pneumococcus. Science 324:1454–1457. doi:10.1126/science.1171908. 19520963

[17] Chaguza C, Cornick JE, Everett DB 2015 Mechanisms and impact of genetic recombination in the evolution of Streptococcus pneumoniae. Comput Struct Biotechnol J 13:241–247. doi:10.1016/j.csbj.2015.03.007. 25904996 PMC4404416

[18] Everett DB, Cornick J, Denis B, Chewapreecha C, Croucher N, Harris S, Parkhill J, Gordon S, Carrol ED, French N, Heyderman RS, Bentley SD 2012 Genetic characterisation of Malawian pneumococci prior to the roll-out of the PCV13 vaccine using a high-throughput whole genome sequencing approach. PLoS One 7:e01053-16. doi:10.1371/journal.pone.0044250. PMC3438182 22970189

[19] Croucher NJ, Harris SR, Fraser C, Quail MA, Burton J, van der Linden M, McGee L, von Gottberg A, Song JH, Ko KS, Pichon B, Baker S, Parry CM, Lambertsen LM, Shahinas D, Pillai DR, Mitchell TJ, Dougan G, Tomasz A, Klugman KP, Parkhill J, Hanage WP, Bentley SD 2011 Rapid pneumococcal evolution in response to clinical interventions. Science 331:430–434. doi:10.1126/science.1198545. 21273480 PMC3648787

[20] Croucher NJ, Finkelstein JA, Pelton SI, Mitchell PK, Lee GM, Parkhill J, Bentley SD, Hanage WP, Lipsitch M 2013 Population genomics of post-vaccine changes in pneumococcal epidemiology. Nat Genet 45:656–663. doi:10.1038/ng.2625. 23644493 PMC3725542

[21] Weinberger DM, Trzciński K, Lu YJ, Bogaert D, Brandes A, Galagan J, Anderson PW, Malley R, Lipsitch M 2009 Pneumococcal capsular polysaccharide structure predicts serotype prevalence. PLoS Pathog 5:e01053-16. doi:10.1371/journal.ppat.1000476. PMC2689349 19521509

[22] Brueggemann AB, Griffiths DT, Meats E, Peto T, Crook DW, Spratt BG 2003 Clonal relationships between invasive and carriage Streptococcus pneumoniae and serotype- and clone-specific differences in invasive disease potential. J Infect Dis 187:1424–1432. doi:10.1086/374624. 12717624

[23] Sleeman KL, Griffiths D, Shackley F, Diggle L, Gupta S, Maiden MC, Moxon ER, Crook DW, Peto TEA 2006 Capsular serotype–specific attack rates and duration of carriage of Streptococcus pneumoniae in a population of children. J Infect Dis 194:682–688. doi:10.1086/505710. 16897668

[24] Hanage WP, Kaijalainen TH, Syrjänen RK, Auranen K, Leinonen M, Mäkelä PH, Spratt BG 2005 Invasiveness of serotypes and clones of Streptococcus pneumoniae among children in Finland. Infect Immun 73:431–435. doi:10.1128/IAI.73.1.431-435.2005. 15618181 PMC538975

[25] Li Y, Weinberger DM, Thompson CM, Trzciński K, Lipsitch M 2013 Surface charge of Streptococcus pneumoniae predicts serotype distribution. Infect Immun 81:4519–4524. doi:10.1128/IAI.00724-13. 24082068 PMC3837974

[26] Chewapreecha C, Harris SR, Croucher NJ, Turner C, Marttinen P, Cheng L, Pessia A, Aanensen DM, Mather AE, Page AJ, Salter SJ, Harris D, Nosten F, Goldblatt D, Corander J, Parkhill J, Turner P, Bentley SD 2014 Dense genomic sampling identifies highways of pneumococcal recombination. Nat Genet 46:305–309. doi:10.1038/ng.2895. 24509479 PMC3970364

[27] Marks LR, Reddinger RM, Hakansson AP 2012 High levels of genetic recombination during nasopharyngeal carriage and biofilm formation in Streptococcus pneumoniae. mBio 3(5):e01053-16. doi:10.1128/mBio.00200-12. PMC3448161 23015736

[28] Andam CP, Hanage WP 2015 Mechanisms of genome evolution of Streptococcus. Infect Genet Evol 33:334–342. doi:10.1016/j.meegid.2014.11.007. 25461843 PMC4430445

[29] Kamng’ona AW, Hinds J, Bar-Zeev N, Gould KA, Chaguza C, Msefula C, Cornick JE, Kulohoma BW, Gray K, Bentley SD, French N, Heyderman RS, Everett DB 2015 High multiple carriage and emergence of Streptococcus pneumoniae vaccine serotype variants in Malawian children. BMC Infect Dis 15:234. doi:10.1186/s12879-015-0980-2. 26088623 PMC4474563

[30] Zerbino DR, Birney E 2008 Velvet: algorithms for de novo short read assembly using de Bruijn graphs. Genome Res 18:821–829. doi:10.1101/gr.074492.107. 18349386 PMC2336801

[31] Zerbino DR 2010 Using the Velvet de novo assembler for short-read sequencing technologies. Curr Protoc Bioinformatics Chapter 11:Unit 11.5. doi:10.1002/0471250953.bi1105s31. PMC2952100 20836074

[32] Page AJ, Cummins CA, Hunt M, Wong VK, Reuter S, Holden MTG, Fookes M, Keane JA, Parkhill J 2015 Roary: rapid large-scale prokaryote pan genome analysis. Bioinformatics 31:3691–3693. doi:10.1093/bioinformatics/btv421. 26198102 PMC4817141

[33] Corander J, Waldmann P, Sillanpää MJ 2003 Bayesian analysis of genetic differentiation between populations. Genetics 163:367–374. 12586722 10.1093/genetics/163.1.367 PMC1462429

[34] Cheng L, Connor TR, Sirén J, Aanensen DM, Corander J 2013 Hierarchical and spatially explicit clustering of DNA sequences with BAPS software. Mol Biol Evol 30:1224–1228. doi:10.1093/molbev/mst028. 23408797 PMC3670731

[35] Croucher NJ, Page AJ, Connor TR, Delaney AJ, Keane JA, Bentley SD, Parkhill J, Harris SR 2015 Rapid phylogenetic analysis of large samples of recombinant bacterial whole genome sequences using Gubbins. Nucleic Acids Res 43:e15. doi:10.1093/nar/gku1196. PMC4330336 25414349

[36] Abdullahi O, Karani A, Tigoi CC, Mugo D, Kungu S, Wanjiru E, Jomo J, Musyimi R, Lipsitch M, Scott JA 2012 Rates of acquisition and clearance of pneumococcal serotypes in the nasopharynges of children in Kilifi District, Kenya. J Infect Dis 206:1020–1029. doi:10.1093/infdis/jis447. 22829650 PMC3433858

[37] Smith T, Lehmann D, Montgomery J, Gratten M, Riley ID, Alpers MP 1993 Acquisition and invasiveness of different serotypes of Streptococcus pneumoniae in young children. Epidemiol Infect 111:27–39. doi:10.1017/S0950268800056648. 8348930 PMC2271190

[38] Cornick JE, Chaguza C, Harris SR, Yalcin F, Senghore M, Kiran AM, Govindpershad S, Ousmane S, Plessis MD, Pluschke G, Ebruke C, McGee L, Sigaùque B, Collard J-M, Antonio M, von Gottberg A, French N, Klugman KP, Heyderman RS, Bentley SD, Everett DB, PAGe Consortium. 2015 Region-specific diversification of the highly virulent serotype 1 Streptococcus pneumoniae. Microb Genomics 1:1–13. doi:10.1099/mgen.0.000027. PMC5320570 28348812

[39] Ritchie ND, Mitchell TJ, Evans TJ 2012 What is different about serotype 1 pneumococci? Future Microbiol 7:33–46. doi:10.2217/fmb.11.146. 22191445

[40] Donkor ES, Bishop CJ, Gould K, Hinds J, Antonio M, Wren B, Hanage WP 2011 High levels of recombination among Streptococcus pneumoniae isolates from the Gambia. mBio 2(3):e01053-16. doi:10.1128/mBio.00040-11. PMC3119534 21693638

[41] Hyams C, Yuste J, Bax K, Camberlein E, Weiser JN, Brown JS 2010 Streptococcus pneumoniae resistance to complement-mediated immunity is dependent on the capsular serotype. Infect Immun 78:716–725. doi:10.1128/IAI.01056-09. 19948838 PMC2812205

[42] Hyams C, Camberlein E, Cohen JM, Bax K, Brown JS 2010 The Streptococcus pneumoniae capsule inhibits complement activity and neutrophil phagocytosis by multiple mechanisms. Infect Immun 78:704–715. doi:10.1128/IAI.00881-09. 19948837 PMC2812187

[43] Page AJ, De Silva N, Hunt M, Quail MA, Parkhill J, Harris SR, Otto TD, Keane JA 2016 Robust high throughput prokaryote *de novo* assembly and improvement pipeline for Illumina data. bioRxiv doi:10.1101/052688. PMC5320598 28348874

[44] Boetzer M, Henkel CV, Jansen HJ, Butler D, Pirovano W 2011 Scaffolding pre-assembled contigs using SSPACE. Bioinformatics 27:578–579. doi:10.1093/bioinformatics/btq683. 21149342

[45] Nadalin F, Vezzi F, Policriti A 2012 GapFiller: a de novo assembly approach to fill the gap within paired reads. BMC Bioinformatics 13:S8. doi:10.1186/1471-2105-13-S14-S8. 23095524 PMC3439727

[46] Stajich JE, Block D, Boulez K, Brenner SE, Chervitz SA, Dagdigian C, Fuellen G, Gilbert JG, Korf I, Lapp H, Lehväslaiho H, Matsalla C, Mungall CJ, Osborne BI, Pocock MR, Schattner P, Senger M, Stein LD, Stupka E, Wilkinson MD, Birney E 2002 The Bioperl toolkit: Perl modules for the life sciences. Genome Res 12:1611–1618. doi:10.1101/gr.361602. 12368254 PMC187536

[47] Croucher NJ, Harris SR, Fraser C, Quail MA, Burton J, van der Linden M, McGee L, von Gottberg A, Song JH, Ko KS, Pichon B, Baker S, Parry CM, Lambertsen LM, Shahinas D, Pillai DR, Mitchell TJ, Dougan G, Tomasz A, Klugman KP, Parkhill J, Hanage WP, Bentley SD 2011 Rapid pneumococcal evolution in response to clinical interventions. Science 331:430–434. doi:10.1126/science.1198545. 21273480 PMC3648787

[48] Stamatakis A 2006 RAxML-VI-HPC: maximum likelihood-based phylogenetic analyses with thousands of taxa and mixed models. Bioinformatics 22:2688–2690. doi:10.1093/bioinformatics/btl446. 16928733

[49] Tavaré S 1986 Some probabilistic and statistical problems in the analysis of DNA sequences, p 57–86. *In* Lectures on mathematics in the life sciences, vol 17 American Mathematical Society, Providence, RI.

[50] Hilty M, Wüthrich D, Salter SJ, Engel H, Campbell S, Sá-Leão R, de Lencastre H, Hermans P, Sadowy E, Turner P, Chewapreecha C, Diggle M, Pluschke G, McGee L, Köseoğlu Eser Ö, Low DE, Smith-Vaughan H, Endimiani A, Küffer M, Dupasquier M, Beaudoing E, Weber J, Bruggmann R, Hanage WP, Parkhill J, Hathaway LJ, Mühlemann K, Bentley SD 2014 Global phylogenomic analysis of nonencapsulated Streptococcus pneumoniae reveals a deep-branching classic lineage that is distinct from multiple sporadic lineages. Genome Biol Evol 6:3281–3294. doi:10.1093/gbe/evu263. 25480686 PMC4986459

[51] Letunic I, Bork P 2011 Interactive Tree of Life v2: online annotation and display of phylogenetic trees made easy. Nucleic Acids Res 39:W475–W478. doi:10.1093/nar/gkr201. 21470960 PMC3125724

[52] Cock PJ, Antao T, Chang JT, Chapman BA, Cox CJ, Dalke A, Friedberg I, Hamelryck T, Kauff F, Wilczynski B, de Hoon MJ 2009 Biopython: freely available Python tools for computational molecular biology and bioinformatics. Bioinformatics 25:1422–1423. doi:10.1093/bioinformatics/btp163. 19304878 PMC2682512

[53] McKenna A, Hanna M, Banks E, Sivachenko A, Cibulskis K, Kernytsky A, Garimella K, Altshuler D, Gabriel S, Daly M, DePristo MA 2010 The Genome Analysis Toolkit: a MapReduce framework for analyzing next-generation DNA sequencing data. Genome Res 20:1297–1303. doi:10.1101/gr.107524.110. 20644199 PMC2928508

