# Supplementary figures and images for: Recombination in Streptococcus pneumoniae Lineages Increase with Carriage Duration and Size of the Polysaccharide Capsule
Source: mBio. 2016 Sep 27;7(5):e01053-16. doi: 10.1128/mBio.01053-16 (PMC5040112; doi:10.1128/mBio.01053-16)

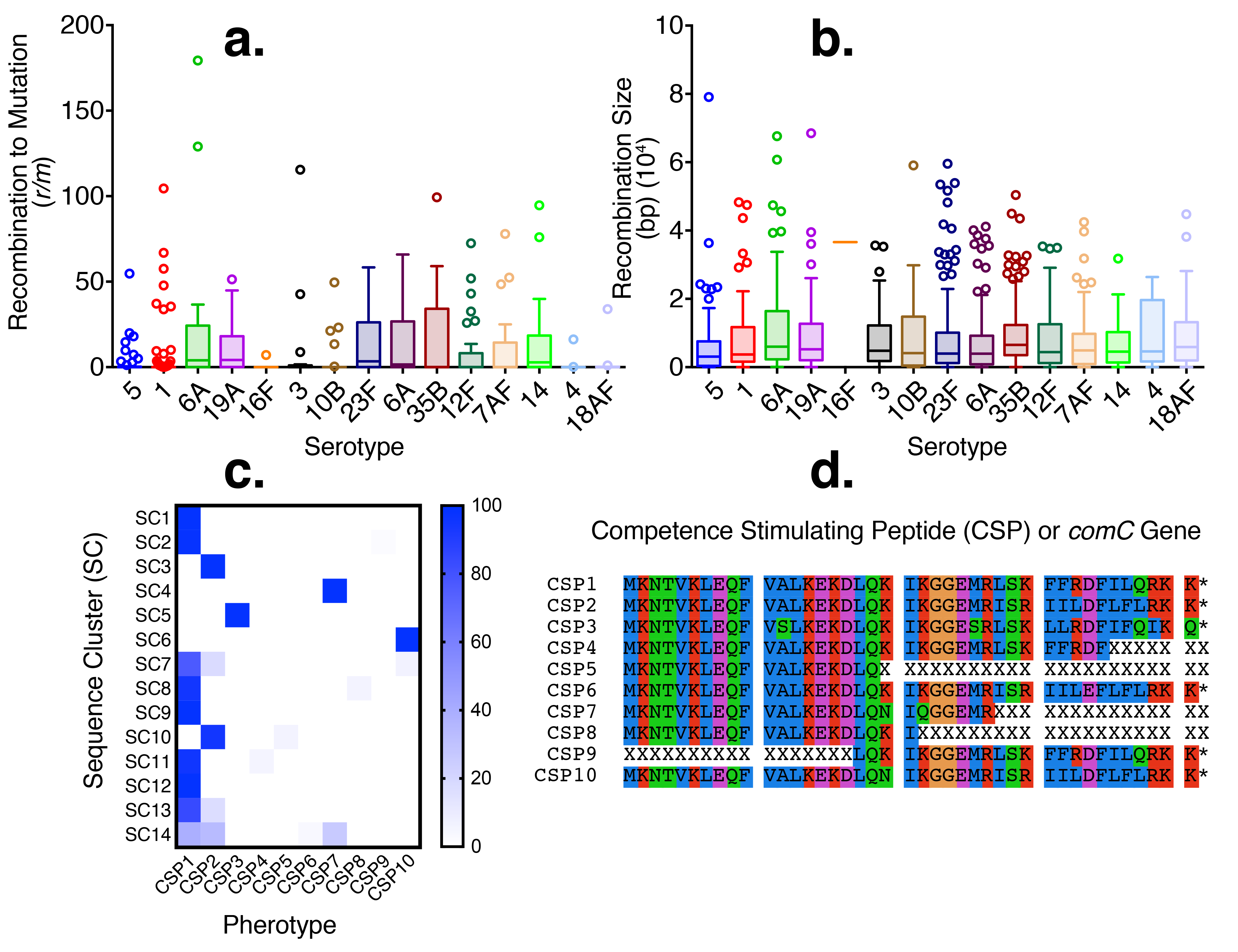

Supplement: Figure S2 — Characteristics of the genetic recombination events identified in the pneumococcal isolates. (a) Distribution of recombination rates on branches of the serotype-specific phylogenies; (b) sizes of recombination blocks identified in the genomes of each pneumococcal serotype; (c) distribution of the competence-stimulating peptide (CSP) variants across the sequence clusters (SCs); (d) protein multiple-sequence alignment of the CSPs encoded by the comC gene. Other variants of the comC gene in additional to the CSP1 and CSP2 were arbitrarily named CPS3 to CSP10. Download [file mbo005163006sf2.tif]

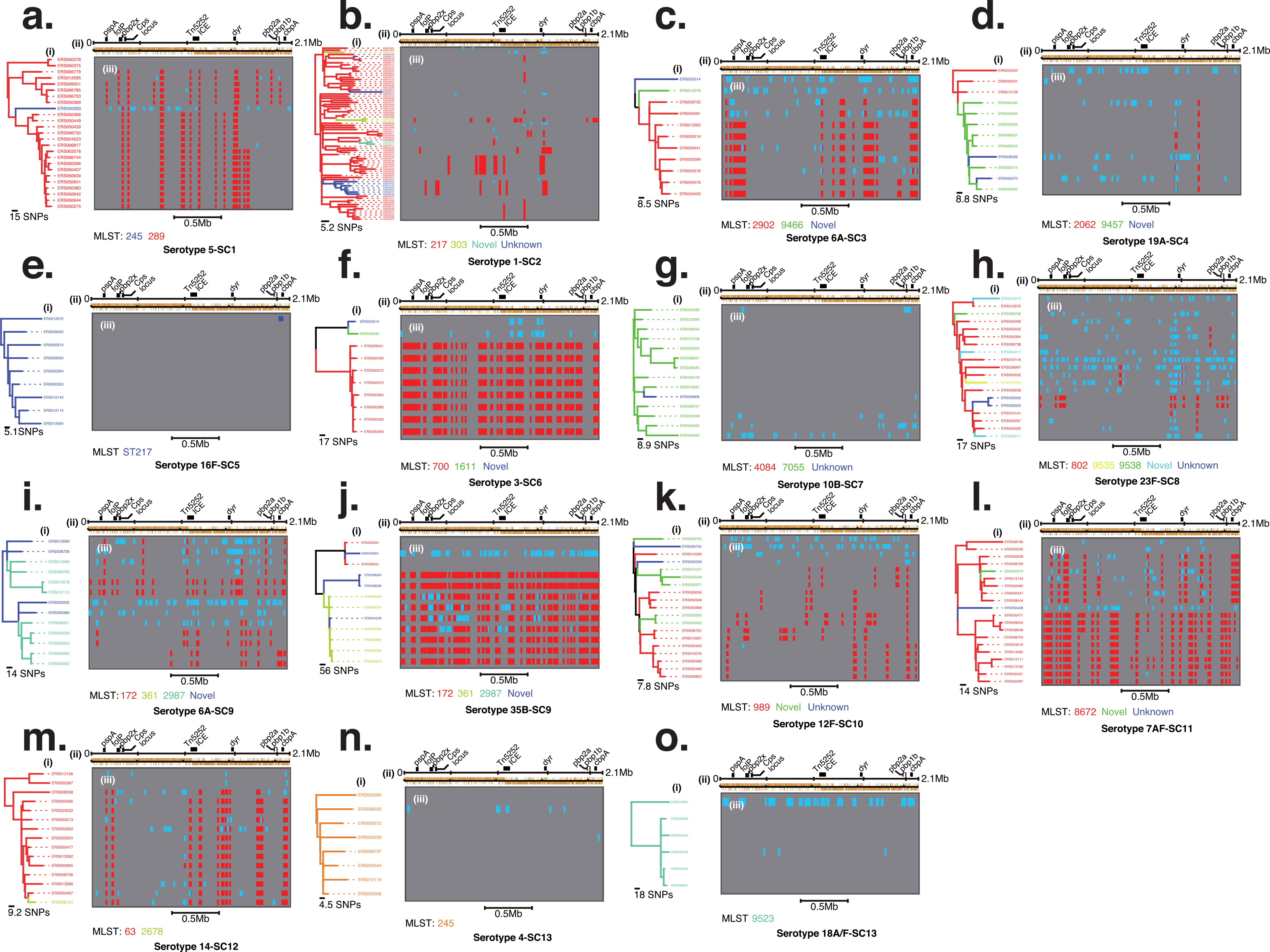

Supplement: Figure S3 — Recombination inferred by Gubbins. Pneumococcal serotypes 5-SC1 (a), 2-SC1 (b), 6A-SC3 (c), 19A-SC4 (d), 16F-SC5 (e), 3-SC6 (f), 10B-SC7 (g), 23F-SC8 (h), 6A-SC9 (i), 35B-SC9 (j), 12F-SC10 (k), 7AF-SC11 (l), 14-SC12 (m), 4-SC13 (n), and 18 A/F-SC13 (o). (i) Maximum likelihood phylogenetic trees of the different serotypes. Branches and tips of the phylogeny are colored according to the MLST sequence type (ST). (ii) Schematic representation of the reference S. pneumoniae genome showing all the genetic annotations and locations of some well-known genes. (iii) Matrix showing the tracks representing each genome. The locations and distribution of regions, which have acquired exogenous DNA through recombination, are colored depending on the number of strains that contain them. Recombination events in internal branches (red) were present in multiple isolates and were shared through clonal descent, rather than independent acquisitions, while those in the terminal branches (blue) were isolate specific and represent independent recent acquisitions. The recombination rate (μr/m), i.e., mean number of the inferred distinct recombination events per isolate (each shared ancestral recombination event that occurred once and spread in the clone via clonal descent was counted once) and the recombination frequency (μre), i.e., the mean number of SNPs introduced through recombination to those introduced through mutation are shown. A high presence of recent recombination events rather than shared recombination events implies a high μre. Download [file mbo005163006sf3.tif]
