# Supplementary material for: Recombination in Streptococcus pneumoniae Lineages Increase with Carriage Duration and Size of the Polysaccharide Capsule
Source: mBio. 2016 Sep 27;7(5):e01053-16. doi: 10.1128/mBio.01053-16 (PMC5040112; doi:10.1128/mBio.01053-16)
Supplement: Table S1 — Streptococcus pneumoniae isolates used in this study. [file mbo005163006st1.docx]

**Table S1:** *S. pneumoniae* strains used in the study

| **Sample ID** | **Sample Accession** | **Run Accession** | **Submission Accession** | **Sequence Cluster (SC)** | **Source** | **Sequence Type (ST)** | **Vaccine Status** | **Serotype** | **Gender** | **Year of Isolation** | **Sequencing Technology** |
| --- | --- | --- | --- | --- | --- | --- | --- | --- | --- | --- | --- |
| D27816 | ERS006730 | ERR018028 | ERA012034 | SC1 | Blood | ST289 | VT | 5 | Female | 2004 | Illumina |
| A28426 | ERS006779 | ERR018081 | ERA012034 | SC1 | Blood | ST289 | VT | 5 | Male | 2004 | Illumina |
| A43327 | ERS006785 | ERR018087 | ERA012034 | SC1 | Blood | ST289 | VT | 5 | Unknown | 2006 | Illumina |
| A43874 | ERS006793 | ERR018096 | ERA012034 | SC1 | Blood | ST289 | VT | 5 | Unknown | 2006 | Illumina |
| D40273 | ERS026051 | ERR033109 | ERA026370 | SC1 | Blood | ST289 | VT | 5 | Male | 2006 | Illumina |
| A29101 | ERS012095 | ERR026614 | ERA015971 | SC1 | Blood | ST289 | VT | 5 | Male | 2004 | Illumina |
| D46845 | ERS050639 | ERR064601 | ERA079165 | SC1 | Blood | ST289 | VT | 5 | Unknown | 2008 | Illumina |
| A37482 | ERS050366 | ERR068207 | ERA080989 | SC1 | Blood | ST289 | VT | 5 | Male | 2005 | Illumina |
| A38608 | ERS050369 | ERR068210 | ERA080989 | SC1 | Blood | ST289 | VT | 5 | Female | 2005 | Illumina |
| A43030 | ERS050375 | ERR068216 | ERA080989 | SC1 | Blood | ST289 | VT | 5 | Unknown | 2006 | Illumina |
| A43904 | ERS050378 | ERR068219 | ERA080989 | SC1 | Blood | ST289 | VT | 5 | Unknown | 2006 | Illumina |
| A44175 | ERS050383 | ERR068224 | ERA080989 | SC1 | Blood | ST289 | VT | 5 | Unknown | 2006 | Illumina |
| A52248 | ERS050393 | ERR068234 | ERA080989 | SC1 | Blood | ST245 | VT | 5 | Female | 2008 | Illumina |
| D48490 | ERS050641 | ERR085862 | ERA087412 | SC1 | Blood | ST289 | VT | 5 | Female | 2008 | Illumina |
| D49319 | ERS050642 | ERR085863 | ERA087412 | SC1 | Blood | ST289 | VT | 5 | Female | 2008 | Illumina |
| D52077 | ERS050644 | ERR085865 | ERA087412 | SC1 | Blood | ST289 | VT | 5 | Male | 2009 | Illumina |
| A59399 | ERS050407 | ERR085958 | ERA087412 | SC1 | Blood | ST289 | VT | 5 | Male | 2010 | Illumina |
| C15085 | ERS006744 | ERR018043 | ERA012034 | SC1 | CSF | ST289 | VT | 5 | Unknown | 2006 | Illumina |
| C16023 | ERS024523 | ERR033150 | ERA026370 | SC1 | CSF | ST289 | VT | 5 | Unknown | 2007 | Illumina |
| C15256 | ERS006817 | ERR023384 | ERA015706 | SC1 | CSF | ST289 | VT | 5 | Female | 2006 | Illumina |
| B16728 | ERS003576 | ERR023984 | ERA015737 | SC1 | CSF | ST289 | VT | 5 | Female | 2007 | Illumina |
| B21609 | ERS050439 | ERR085990 | ERA087412 | SC1 | CSF | ST289 | VT | 5 | Female | 2010 | Illumina |
| B9517 | ERS050449 | ERR086000 | ERA087412 | SC1 | CSF | ST289 | VT | 5 | Male | 2004 | Illumina |
| BAN1HP | ERS050266 | ERR068108 | ERA080989 | SC1 | Carriage | ST289 | VT | 5 | Male | 2011 | Illumina |
| BAN1JL | ERS050275 | ERR068117 | ERA080989 | SC1 | Carriage | ST289 | VT | 5 | Female | 2011 | Illumina |
| D33521 | ERS006728 | ERR018026 | ERA012034 | SC2 | Blood | ST217 | VT | 1 | Male | 2005 | Illumina |
| A41093 | ERS006774 | ERR018075 | ERA012034 | SC2 | Blood | ST217~ | VT | 1 | Unknown | 2006 | Illumina |
| A37371 | ERS006772 | ERR018076 | ERA012034 | SC2 | Blood | ST217 | VT | 1 | Female | 2005 | Illumina |
| A41626 | ERS006783 | ERR018085 | ERA012034 | SC2 | Blood | ST217 | VT | 1 | Unknown | 2006 | Illumina |
| A35070 | ERS006787 | ERR018090 | ERA012034 | SC2 | Blood | ST217 | VT | 1 | Male | 2005 | Illumina |
| A30928 | ERS006788 | ERR018091 | ERA012034 | SC2 | Blood | ST217 | VT | 1 | Male | 2004 | Illumina |
| A30338 | ERS006789 | ERR018092 | ERA012034 | SC2 | Blood | ST217 | VT | 1 | Male | 2004 | Illumina |
| A46444 | ERS006799 | ERR018103 | ERA012034 | SC2 | Blood | ST217 | VT | 1 | Male | 2007 | Illumina |
| D42299 | ERS026060 | ERR033114 | ERA026370 | SC2 | Blood | ST217 | VT | 1 | Male | 2007 | Illumina |
| D25796 | ERS024510 | ERR033122 | ERA026370 | SC2 | Blood | ST217 | VT | 1 | Female | 2004 | Illumina |
| D34485 | ERS024512 | ERR033123 | ERA026370 | SC2 | Blood | ST217 | VT | 1 | Female | 2005 | Illumina |
| D28981 | ERS024516 | ERR033130 | ERA026370 | SC2 | Blood | ST217 | VT | 1 | Unknown | 2004 | Illumina |
| D38643 | ERS026042 | ERR033146 | ERA026370 | SC2 | Blood | ST217 | VT | 1 | Female | 2006 | Illumina |
| D48309 | ERS024531 | ERR033153 | ERA026370 | SC2 | Blood | ST217 | VT | 1 | Female | 2008 | Illumina |
| A55084 | ERS006809 | ERR023376 | ERA015706 | SC2 | Blood | ST217 | VT | 1 | Female | 2009 | Illumina |
| A55122 | ERS006811 | ERR023378 | ERA015706 | SC2 | Blood | ST217 | VT | 1 | Male | 2009 | Illumina |
| A36633 | ERS006825 | ERR023394 | ERA015706 | SC2 | Blood | ST217 | VT | 1 | Female | 2005 | Illumina |
| A33056 | ERS003550 | ERR023964 | ERA015737 | SC2 | Blood | ST303 | VT | 1 | Male | 2005 | Illumina |
| A49293 | ERS003573 | ERR023981 | ERA015737 | SC2 | Blood | ST217 | VT | 1 | Male | 2007 | Illumina |
| A34978 | ERS008321 | ERR024497 | ERA015791 | SC2 | Blood | ST217 | VT | 1 | Unknown | 2005 | Illumina |
| A35711 | ERS008323 | ERR024499 | ERA015791 | SC2 | Blood | ST11745 | VT | 1 | Female | 2005 | Illumina |
| A37104 | ERS008326 | ERR024502 | ERA015791 | SC2 | Blood | ST217 | VT | 1 | Male | 2005 | Illumina |
| D40696 | ERS008363 | ERR024537 | ERA015791 | SC2 | Blood | ST217 | VT | 1 | Male | 2007 | Illumina |
| A39247 | ERS012061 | ERR028396 | ERA020597 | SC2 | Blood | ST217 | VT | 1 | Unknown | 2006 | Illumina |
| A41267 | ERS012063 | ERR028400 | ERA020597 | SC2 | Blood | ST217 | VT | 1 | Unknown | 2006 | Illumina |
| A42174 | ERS012069 | ERR028406 | ERA020597 | SC2 | Blood | ST217 | VT | 1 | Unknown | 2006 | Illumina |
| A28816 | ERS012093 | ERR028422 | ERA020597 | SC2 | Blood | ST217 | VT | 1 | Male | 2004 | Illumina |
| A33973 | ERS012120 | ERR028438 | ERA020597 | SC2 | Blood | ST217 | VT | 1 | Female | 2005 | Illumina |
| A34030 | ERS012121 | ERR028439 | ERA020597 | SC2 | Blood | ST217 | VT | 1 | Unknown | 2005 | Illumina |
| A34045 | ERS012122 | ERR028440 | ERA020597 | SC2 | Blood | ST11745 | VT | 1 | Female | 2005 | Illumina |
| A35279 | ERS012127 | ERR028444 | ERA020597 | SC2 | Blood | ST217 | VT | 1 | Female | 2005 | Illumina |
| A36780 | ERS012129 | ERR028447 | ERA020597 | SC2 | Blood | ST11745 | VT | 1 | Male | 2005 | Illumina |
| A31105 | ERS050335 | ERR068176 | ERA080989 | SC2 | Blood | ST217 | VT | 1 | Male | 2004 | Illumina |
| A31584 | ERS050342 | ERR068183 | ERA080989 | SC2 | Blood | ST217 | VT | 1 | Female | 2004 | Illumina |
| A32572 | ERS050346 | ERR068187 | ERA080989 | SC2 | Blood | ST217 | VT | 1 | Female | 2004 | Illumina |
| A35715 | ERS050350 | ERR068191 | ERA080989 | SC2 | Blood | ST217 | VT | 1 | Male | 2005 | Illumina |
| A37004 | ERS050361 | ERR068202 | ERA080989 | SC2 | Blood | ST217 | VT | 1 | Female | 2005 | Illumina |
| A37046 | ERS050362 | ERR068203 | ERA080989 | SC2 | Blood | ST217 | VT | 1 | Female | 2005 | Illumina |
| A37089 | ERS050363 | ERR068204 | ERA080989 | SC2 | Blood | ST217 | VT | 1 | Female | 2005 | Illumina |
| A43126 | ERS050376 | ERR068217 | ERA080989 | SC2 | Blood | ST217 | VT | 1 | Unknown | 2006 | Illumina |
| A44013 | ERS050379 | ERR068220 | ERA080989 | SC2 | Blood | ST217 | VT | 1 | Unknown | 2006 | Illumina |
| A44026 | ERS050380 | ERR068221 | ERA080989 | SC2 | Blood | ST217 | VT | 1 | Unknown | 2006 | Illumina |
| A44049 | ERS050381 | ERR068222 | ERA080989 | SC2 | Blood | ST217 | VT | 1 | Unknown | 2006 | Illumina |
| A44163 | ERS050382 | ERR068223 | ERA080989 | SC2 | Blood | ST217 | VT | 1 | Unknown | 2006 | Illumina |
| A51425 | ERS050388 | ERR068229 | ERA080989 | SC2 | Blood | ST217 | VT | 1 | Female | 2008 | Illumina |
| A53108 | ERS050395 | ERR068236 | ERA080989 | SC2 | Blood | ST217 | VT | 1 | Male | 2008 | Illumina |
| A53532 | ERS050397 | ERR068238 | ERA080989 | SC2 | Blood | ST11745 | VT | 1 | Male | 2008 | Illumina |
| D45902 | ERS050638 | ERR085859 | ERA087412 | SC2 | Blood | ST217 | VT | 1 | Female | 2008 | Illumina |
| A58264 | ERS050405 | ERR085956 | ERA087412 | SC2 | Blood | ST217 | VT | 1 | Female | 2010 | Illumina |
| A59539 | ERS050408 | ERR085959 | ERA087412 | SC2 | Blood | ST217~ | VT | 1 | Male | 2010 | Illumina |
| D27102 | ERS050494 | ERR086045 | ERA087412 | SC2 | Blood | ST11745 | VT | 1 | Female | 2004 | Illumina |
| D35438 | ERS050496 | ERR086047 | ERA087412 | SC2 | Blood | ST217 | VT | 1 | Female | 2005 | Illumina |
| C9471 | ERS006723 | ERR018020 | ERA012034 | SC2 | CSF | ST217 | VT | 1 | Unknown | 2004 | Illumina |
| C14099 | ERS006734 | ERR018021 | ERA012034 | SC2 | CSF | ST217 | VT | 1 | Female | 2006 | Illumina |
| C14940 | ERS006731 | ERR018029 | ERA012034 | SC2 | CSF | ST11745 | VT | 1 | Male | 2006 | Illumina |
| C13181 | ERS006746 | ERR018034 | ERA012034 | SC2 | CSF | ST217 | VT | 1 | Female | 2005 | Illumina |
| C16000 | ERS006743 | ERR018042 | ERA012034 | SC2 | CSF | ST217 | VT | 1 | Male | 2007 | Illumina |
| C13215 | ERS006745 | ERR018044 | ERA012034 | SC2 | CSF | ST217 | VT | 1 | Female | 2005 | Illumina |
| B17447 | ERS006803 | ERR018107 | ERA012034 | SC2 | CSF | ST217 | VT | 1 | Male | 2008 | Illumina |
| C18031 | ERS026063 | ERR033151 | ERA026370 | SC2 | CSF | ST217 | VT | 1 | Male | 2008 | Illumina |
| C19609 | ERS006814 | ERR023381 | ERA015706 | SC2 | CSF | ST217 | VT | 1 | Male | 2008 | Illumina |
| C14249 | ERS006820 | ERR023389 | ERA015706 | SC2 | CSF | ST217 | VT | 1 | Male | 2006 | Illumina |
| C11020 | ERS006824 | ERR023393 | ERA015706 | SC2 | CSF | ST303 | VT | 1 | Unknown | 2005 | Illumina |
| B16942 | ERS003577 | ERR023976 | ERA015737 | SC2 | CSF | ST217 | VT | 1 | Male | 2007 | Illumina |
| B17099 | ERS003569 | ERR023977 | ERA015737 | SC2 | CSF | ST217 | VT | 1 | Male | 2007 | Illumina |
| B18718 | ERS003586 | ERR023995 | ERA015737 | SC2 | CSF | ST303 | VT | 1 | Female | 2008 | Illumina |
| B12423 | ERS008341 | ERR024507 | ERA015791 | SC2 | CSF | ST217 | VT | 1 | Female | 2005 | Illumina |
| C15514 | ERS008357 | ERR024531 | ERA015791 | SC2 | CSF | ST217 | VT | 1 | Male | 2007 | Illumina |
| B14935 | ERS012073 | ERR028411 | ERA020597 | SC2 | CSF | ST217 | VT | 1 | Male | 2006 | Illumina |
| B15976 | ERS012084 | ERR028423 | ERA020597 | SC2 | CSF | ST217 | VT | 1 | Male | 2007 | Illumina |
| B17333 | ERS012088 | ERR028427 | ERA020597 | SC2 | CSF | ST217 | VT | 1 | Male | 2008 | Illumina |
| B11467 | ERS012133 | ERR028451 | ERA020597 | SC2 | CSF | ST217 | VT | 1 | Female | 2005 | Illumina |
| B11505 | ERS012134 | ERR028452 | ERA020597 | SC2 | CSF | ST217 | VT | 1 | Female | 2005 | Illumina |
| B12027 | ERS050413 | ERR085964 | ERA087412 | SC2 | CSF | ST217 | VT | 1 | Male | 2005 | Illumina |
| B17224 | ERS050414 | ERR085965 | ERA087412 | SC2 | CSF | ST217 | VT | 1 | Female | 2007 | Illumina |
| B17789 | ERS050415 | ERR085966 | ERA087412 | SC2 | CSF | ST217 | VT | 1 | Male | 2008 | Illumina |
| B17998 | ERS050416 | ERR085967 | ERA087412 | SC2 | CSF | ST217 | VT | 1 | Male | 2008 | Illumina |
| B18042 | ERS050417 | ERR085968 | ERA087412 | SC2 | CSF | ST217 | VT | 1 | Female | 2008 | Illumina |
| C12797 | ERS050453 | ERR086004 | ERA087412 | SC2 | CSF | ST217 | VT | 1 | Female | 2005 | Illumina |
| C21127 | ERS050480 | ERR086031 | ERA087412 | SC2 | CSF | ST11745 | VT | 1 | Male | 2009 | Illumina |
| BAN1C2 | ERS050244 | ERR068086 | ERA080989 | SC2 | Carriage | ST217 | VT | 1 | Female | 2011 | Illumina |
| BAN17G | ERS050217 | ERR068059 | ERA080989 | SC2 | Carriage | ST217~ | VT | 1 | Male | 2011 | Illumina |
| BAN1WT | ERS050313 | ERR068154 | ERA080989 | SC2 | Carriage | ST217~ | VT | 1 | Male | 2011 | Illumina |
| D38094 | ERS006733 | ERR018031 | ERA012034 | SC3 | Blood | ST2902 | VT | 6A | Male | 2006 | Illumina |
| A51131 | ERS003578 | ERR023986 | ERA015737 | SC3 | Blood | ST2902 | VT | 6A | Male | 2008 | Illumina |
| D38023 | ERS012079 | ERR028417 | ERA020597 | SC3 | Blood | ST9466 | VT | 6A | Male | 2006 | Illumina |
| A53886 | ERS050399 | ERR068240 | ERA080989 | SC3 | Blood | ST2902 | VT | 6A | Male | 2009 | Illumina |
| A54130 | ERS050400 | ERR068241 | ERA080989 | SC3 | Blood | ST2902 | VT | 6A | Male | 2009 | Illumina |
| B15901 | ERS012083 | ERR028420 | ERA020597 | SC3 | CSF | ST2902 | VT | 6A | Female | 2007 | Illumina |
| B21980 | ERS050441 | ERR085992 | ERA087412 | SC3 | CSF | ST2902 | VT | 6A | Male | 2010 | Illumina |
| C21075 | ERS050478 | ERR086029 | ERA087412 | SC3 | CSF | ST2902 | VT | 6A | Male | 2009 | Illumina |
| C22644 | ERS050481 | ERR086032 | ERA087412 | SC3 | CSF | ST2902 | VT | 6A | Female | 2010 | Illumina |
| BAN17U | ERS050218 | ERR068060 | ERA080989 | SC3 | Carriage | ST2902 | VT | 6A | Male | 2011 | Illumina |
| BAN209 | ERS050314 | ERR068155 | ERA080989 | SC3 | Carriage | ST2902~ | VT | 6A | Male | 2011 | Illumina |
| A40931 | ERS008329 | ERR024494 | ERA015791 | SC4 | Blood | ST7898~ | VT | 19A | Unknown | 2006 | Illumina |
| A35432 | ERS008322 | ERR024498 | ERA015791 | SC4 | Blood | ST9457 | VT | 19A | Female | 2005 | Illumina |
| A36652 | ERS012128 | ERR028446 | ERA020597 | SC4 | Blood | ST2062 | VT | 19A | Female | 2005 | Illumina |
| A31349 | ERS050340 | ERR068181 | ERA080989 | SC4 | Blood | ST9457 | VT | 19A | Female | 2004 | Illumina |
| A31361 | ERS050341 | ERR068182 | ERA080989 | SC4 | Blood | ST2062 | VT | 19A | Female | 2004 | Illumina |
| A35786 | ERS050352 | ERR068193 | ERA080989 | SC4 | Blood | ST2062 | VT | 19A | Female | 2005 | Illumina |
| A36173 | ERS050355 | ERR068196 | ERA080989 | SC4 | Blood | ST9457 | VT | 19A | Male | 2005 | Illumina |
| A37452 | ERS050365 | ERR068206 | ERA080989 | SC4 | Blood | ST9457 | VT | 19A | Female | 2005 | Illumina |
| B11684 | ERS008337 | ERR024514 | ERA015791 | SC4 | CSF | ST9457 | VT | 19A | Male | 2005 | Illumina |
| C20833 | ERS050474 | ERR086025 | ERA087412 | SC4 | CSF | ST9457 | VT | 19A | Male | 2009 | Illumina |
| BAN1I9 | ERS050270 | ERR068112 | ERA080989 | SC4 | Carriage | ST9457~ | VT | 19A | Male | 2011 | Illumina |
| BAN1Q4 | ERS050284 | ERR068126 | ERA080989 | SC4 | Carriage | ST9457 | VT | 19A | Male | 2011 | Illumina |
| D40857 | ERS026053 | ERR033110 | ERA026370 | SC5 | Blood | ST705 | NVT | 16F | Female | 2007 | Illumina |
| D37909 | ERS026050 | ERR033138 | ERA026370 | SC5 | Blood | ST705 | NVT | 16F | Female | 2006 | Illumina |
| A33075 | ERS012115 | ERR026625 | ERA015971 | SC5 | Blood | ST705 | NVT | 16F | Male | 2005 | Illumina |
| A42190 | ERS012070 | ERR028397 | ERA020597 | SC5 | Blood | ST705 | NVT | 16F | Unknown | 2006 | Illumina |
| A41312 | ERS012064 | ERR028401 | ERA020597 | SC5 | Blood | ST705 | NVT | 16F | Unknown | 2006 | Illumina |
| D30716 | ERS012142 | ERR028461 | ERA020597 | SC5 | Blood | ST705 | NVT | 16F | Unknown | 2005 | Illumina |
| A35997 | ERS050353 | ERR068194 | ERA080989 | SC5 | Blood | ST705 | NVT | 16F | Female | 2005 | Illumina |
| A36147 | ERS050354 | ERR068195 | ERA080989 | SC5 | Blood | ST705 | NVT | 16F | Male | 2005 | Illumina |
| BAN1JK | ERS050274 | ERR068116 | ERA080989 | SC5 | Carriage | ST705 | NVT | 16F | Male | 2011 | Illumina |
| D23908 | ERS024514 | ERR033132 | ERA026370 | SC6 | Blood | ST230~ | VT | 19A | Unknown | 2004 | Illumina |
| D36881 | ERS026041 | ERR033136 | ERA026370 | SC6 | Blood | ST700 | VT | 3 | Male | 2006 | Illumina |
| A35264 | ERS050349 | ERR068190 | ERA080989 | SC6 | Blood | ST700 | VT | 3 | Male | 2005 | Illumina |
| A40924 | ERS050370 | ERR068211 | ERA080989 | SC6 | Blood | ST700 | VT | 3 | Unknown | 2006 | Illumina |
| A52402 | ERS050394 | ERR068235 | ERA080989 | SC6 | Blood | ST700 | VT | 3 | Female | 2008 | Illumina |
| D49759 | ERS050643 | ERR085864 | ERA087412 | SC6 | Blood | ST1611 | VT | 19A | Female | 2009 | Illumina |
| B20539 | ERS050432 | ERR085983 | ERA087412 | SC6 | CSF | ST700 | VT | 3 | Female | 2009 | Illumina |
| B20605 | ERS050433 | ERR085984 | ERA087412 | SC6 | CSF | ST700 | VT | 3 | Male | 2009 | Illumina |
| BAN156 | ERS050212 | ERR068054 | ERA080989 | SC6 | Carriage | ST700 | VT | 3 | Female | 2011 | Illumina |
| BAN1MH | ERS050280 | ERR068122 | ERA080989 | SC6 | Carriage | ST700 | VT | 3 | Male | 2011 | Illumina |
| D31621 | ERS006727 | ERR018025 | ERA012034 | SC7 | Blood | ST7055 | NVT | 10B | Unknown | 2005 | Illumina |
| A42964 | ERS006806 | ERR018099 | ERA012034 | SC7 | Blood | ST7055~ | NVT | 10B | Unknown | 2006 | Illumina |
| A42347 | ERS008331 | ERR024505 | ERA015791 | SC7 | Blood | ST7055 | NVT | 10B | Unknown | 2006 | Illumina |
| A29037 | ERS012094 | ERR026611 | ERA015971 | SC7 | Blood | ST7055 | NVT | 10B | Male | 2004 | Illumina |
| A34568 | ERS012125 | ERR028433 | ERA020597 | SC7 | Blood | ST4084 | NVT | 33D | Female | 2005 | Illumina |
| A34857 | ERS050348 | ERR068189 | ERA080989 | SC7 | Blood | ST7055 | NVT | 10B | Male | 2005 | Illumina |
| A51944 | ERS050390 | ERR068231 | ERA080989 | SC7 | Blood | ST7055 | NVT | 10B | Male | 2008 | Illumina |
| A53112 | ERS050396 | ERR068237 | ERA080989 | SC7 | Blood | ST7055 | NVT | 10B | Female | 2008 | Illumina |
| C11367 | ERS026044 | ERR033144 | ERA026370 | SC7 | CSF | ST7055 | NVT | 10B | Unknown | 2005 | Illumina |
| B11601 | ERS003555 | ERR023972 | ERA015737 | SC7 | CSF | ST7055 | NVT | 10B | Male | 2005 | Illumina |
| B14457 | ERS008345 | ERR024523 | ERA015791 | SC7 | CSF | ST7055 | NVT | 10B | Male | 2006 | Illumina |
| B9399 | ERS012106 | ERR026626 | ERA015971 | SC7 | CSF | ST7055 | NVT | 10B | Male | 2004 | Illumina |
| B16827 | ERS012087 | ERR028426 | ERA020597 | SC7 | CSF | ST7055 | NVT | 10B | Male | 2007 | Illumina |
| B18107 | ERS050418 | ERR085969 | ERA087412 | SC7 | CSF | ST7055 | NVT | 10B | Female | 2008 | Illumina |
| C18320 | ERS050462 | ERR086013 | ERA087412 | SC7 | CSF | ST4084 | NVT | 33D | Female | 2008 | Illumina |
| BAN1BN | ERS050239 | ERR068081 | ERA080989 | SC7 | Carriage | ST4084 | NVT | 33D | Female | 2011 | Illumina |
| BAN1RA | ERS050292 | ERR068134 | ERA080989 | SC7 | Carriage | ST7055 | NVT | 10B | Female | 2011 | Illumina |
| D26316 | ERS006738 | ERR018036 | ERA012034 | SC8 | Blood | ST802 | VT | 23F | Male | 2004 | Illumina |
| D43782 | ERS006758 | ERR018047 | ERA012034 | SC8 | Blood | ST9538 | VT | 23F | Female | 2007 | Illumina |
| D42660 | ERS026061 | ERR033113 | ERA026370 | SC8 | Blood | ST802 | VT | 23F | Female | 2007 | Illumina |
| D46897 | ERS024529 | ERR033155 | ERA026370 | SC8 | Blood | ST9535 | VT | 23F | Female | 2008 | Illumina |
| A36756 | ERS003552 | ERR023969 | ERA015737 | SC8 | Blood | ST802 | VT | 23F | Female | 2005 | Illumina |
| A32273 | ERS008320 | ERR024496 | ERA015791 | SC8 | Blood | ST802~ | VT | 23F | Female | 2004 | Illumina |
| A33308 | ERS012116 | ERR028432 | ERA020597 | SC8 | Blood | ST802 | VT | 23F | Female | 2005 | Illumina |
| D30625 | ERS012141 | ERR028460 | ERA020597 | SC8 | Blood | ST802 | VT | 23F | Unknown | 2005 | Illumina |
| A37297 | ERS050364 | ERR068205 | ERA080989 | SC8 | Blood | ST802 | VT | 23F | Female | 2005 | Illumina |
| C15291 | ERS026058 | ERR033116 | ERA026370 | SC8 | CSF | ST802 | VT | 23F | Female | 2006 | Illumina |
| B10366 | ERS008335 | ERR024512 | ERA015791 | SC8 | CSF | ST802~ | VT | 23F | Male | 2004 | Illumina |
| B14767 | ERS012072 | ERR028408 | ERA020597 | SC8 | CSF | ST802~ | VT | 23F | Male | 2006 | Illumina |
| B15188 | ERS012075 | ERR028413 | ERA020597 | SC8 | CSF | ST802 | VT | 23F | Female | 2006 | Illumina |
| B11055 | ERS050411 | ERR085962 | ERA087412 | SC8 | CSF | ST802~ | VT | 23F | Female | 2005 | Illumina |
| B18340 | ERS050420 | ERR085971 | ERA087412 | SC8 | CSF | ST802 | VT | 23F | Male | 2008 | Illumina |
| B18678 | ERS050423 | ERR085974 | ERA087412 | SC8 | CSF | ST802 | VT | 23F | Male | 2008 | Illumina |
| C17952 | ERS050459 | ERR086010 | ERA087412 | SC8 | CSF | ST802 | VT | 23F | Female | 2008 | Illumina |
| BAN1ID | ERS050271 | ERR068113 | ERA080989 | SC8 | Carriage | ST802~ | VT | 23F | Female | 2011 | Illumina |
| BAN1S4 | ERS050297 | ERR068139 | ERA080989 | SC8 | Carriage | ST802 | VT | 23F | Male | 2011 | Illumina |
| D37725 | ERS006726 | ERR018023 | ERA012034 | SC9 | Blood | ST2987~ | VT | 6A | Male | 2006 | Illumina |
| D47335 | ERS006754 | ERR018054 | ERA012034 | SC9 | Blood | ST361 | NVT | 35B | Female | 2008 | Illumina |
| A16898 | ERS006792 | ERR018095 | ERA012034 | SC9 | Blood | ST2987 | VT | 6A | Unknown | 2002 | Illumina |
| D41212 | ERS026059 | ERR033115 | ERA026370 | SC9 | Blood | ST172 | NVT | 15B/C | Female | 2007 | Illumina |
| D46120 | ERS024528 | ERR033156 | ERA026370 | SC9 | Blood | ST10599 | VT | 19A | Male | 2008 | Illumina |
| A37893 | ERS008328 | ERR024493 | ERA015791 | SC9 | Blood | ST2987 | VT | 6A | Male | 2005 | Illumina |
| A35792 | ERS008324 | ERR024500 | ERA015791 | SC9 | Blood | ST11715 | VT | 19A | Female | 2005 | Illumina |
| D33293 | ERS008360 | ERR024534 | ERA015791 | SC9 | Blood | ST2987 | VT | 6A | Unknown | 2005 | Illumina |
| D24847 | ERS012112 | ERR026632 | ERA015971 | SC9 | Blood | ST2987 | VT | 6A | Female | 2004 | Illumina |
| A42000 | ERS012068 | ERR028405 | ERA020597 | SC9 | Blood | ST2987 | VT | 6A | Unknown | 2006 | Illumina |
| D46329 | ERS012090 | ERR028429 | ERA020597 | SC9 | Blood | ST2987~ | VT | 6A | Female | 2008 | Illumina |
| A36529 | ERS050356 | ERR068197 | ERA080989 | SC9 | Blood | ST172 | NVT | 15B/C | Female | 2005 | Illumina |
| A36891 | ERS050360 | ERR068201 | ERA080989 | SC9 | Blood | ST10596 | VT | 6A | Female | 2005 | Illumina |
| B11856 | ERS008339 | ERR024516 | ERA015791 | SC9 | CSF | ST11715 | VT | 19A | Male | 2005 | Illumina |
| B12772 | ERS008343 | ERR024518 | ERA015791 | SC9 | CSF | ST2987 | VT | 6A | Female | 2005 | Illumina |
| C12495 | ERS008352 | ERR024519 | ERA015791 | SC9 | CSF | ST2987 | VT | 6A | Female | 2005 | Illumina |
| C11950 | ERS008351 | ERR024529 | ERA015791 | SC9 | CSF | ST2987 | VT | 6A | Unknown | 2005 | Illumina |
| C14215 | ERS012078 | ERR028416 | ERA020597 | SC9 | CSF | ST2987 | VT | 6A | Male | 2006 | Illumina |
| B19661 | ERS050426 | ERR085977 | ERA087412 | SC9 | CSF | ST361 | VT | 19F | Male | 2009 | Illumina |
| BAN196 | ERS050225 | ERR068067 | ERA080989 | SC9 | Carriage | ST111~ | VT | 6A | Female | 2011 | Illumina |
| BAN1B6 | ERS050236 | ERR068078 | ERA080989 | SC9 | Carriage | ST361 | NVT | 35B | Female | 2011 | Illumina |
| BAN1E4 | ERS050250 | ERR068092 | ERA080989 | SC9 | Carriage | ST361 | NVT | 35B | Male | 2011 | Illumina |
| BAN1GV | ERS050263 | ERR068105 | ERA080989 | SC9 | Carriage | ST172~ | NVT | 15B/C | Male | 2011 | Illumina |
| BAN1J0 | ERS050273 | ERR068115 | ERA080989 | SC9 | Carriage | ST361 | NVT | 35B | Female | 2011 | Illumina |
| BAN1ES | ERS050922 | ERR068243 | ERA080989 | SC9 | Carriage | ST361 | NVT | 35B | Male | 2011 | Illumina |
| D38612 | ERS006755 | ERR018055 | ERA012034 | SC10 | Blood | ST6839~ | NVT | 12F | Female | 2006 | Illumina |
| A29943 | ERS012097 | ERR026616 | ERA015971 | SC10 | Blood | ST989 | NVT | 12B | Female | 2004 | Illumina |
| A31131 | ERS012099 | ERR026618 | ERA015971 | SC10 | Blood | ST989 | NVT | 12F | Male | 2004 | Illumina |
| A31198 | ERS050338 | ERR068179 | ERA080989 | SC10 | Blood | ST989 | NVT | 12F | Female | 2004 | Illumina |
| A36782 | ERS050359 | ERR068200 | ERA080989 | SC10 | Blood | ST989~ | NVT | 12F | Female | 2005 | Illumina |
| A37837 | ERS050368 | ERR068209 | ERA080989 | SC10 | Blood | ST989 | NVT | 12F | Female | 2005 | Illumina |
| A43416 | ERS050377 | ERR068218 | ERA080989 | SC10 | Blood | ST989~ | NVT | 12F | Unknown | 2006 | Illumina |
| A52232 | ERS050392 | ERR068233 | ERA080989 | SC10 | Blood | ST989~ | NVT | 12F | Male | 2008 | Illumina |
| A56826 | ERS050403 | ERR085954 | ERA087412 | SC10 | Blood | ST989 | NVT | 12F | Male | 2009 | Illumina |
| C9536 | ERS006725 | ERR018022 | ERA012034 | SC10 | CSF | ST989 | NVT | 12B | Male | 2004 | Illumina |
| C18004 | ERS006740 | ERR018039 | ERA012034 | SC10 | CSF | ST11780 | NVT | 12A | Male | 2008 | Illumina |
| B17731 | ERS006802 | ERR018106 | ERA012034 | SC10 | CSF | ST989 | NVT | 12F | Female | 2008 | Illumina |
| C15284 | ERS026056 | ERR033118 | ERA026370 | SC10 | CSF | ST989 | NVT | 12F | Unknown | 2006 | Illumina |
| B13048 | ERS003548 | ERR023966 | ERA015737 | SC10 | CSF | ST989~ | NVT | 12F | Male | 2005 | Illumina |
| B9437 | ERS012107 | ERR026627 | ERA015971 | SC10 | CSF | ST989~ | NVT | 12F | Male | 2004 | Illumina |
| B15249 | ERS012076 | ERR028414 | ERA020597 | SC10 | CSF | ST989 | NVT | 12F | Female | 2006 | Illumina |
| B18494 | ERS050422 | ERR085973 | ERA087412 | SC10 | CSF | ST989~ | NVT | 12F | Male | 2008 | Illumina |
| C18919 | ERS050466 | ERR086017 | ERA087412 | SC10 | CSF | ST989 | NVT | 12F | Female | 2008 | Illumina |
| C19054 | ERS050469 | ERR086020 | ERA087412 | SC10 | CSF | ST989 | NVT | 12F | Male | 2008 | Illumina |
| D31870 | ERS006729 | ERR018027 | ERA012034 | SC11 | Blood | ST8672 | VT | 7A/F | Unknown | 2005 | Illumina |
| D47367 | ERS006753 | ERR018053 | ERA012034 | SC11 | Blood | ST8672 | VT | 7A/F | Male | 2008 | Illumina |
| A49600 | ERS006796 | ERR018102 | ERA012034 | SC11 | Blood | ST8672 | VT | 7A/F | Female | 2008 | Illumina |
| A52018 | ERS008334 | ERR024511 | ERA015791 | SC11 | Blood | ST8672 | VT | 7A/F | Male | 2008 | Illumina |
| D23780 | ERS012111 | ERR026631 | ERA015971 | SC11 | Blood | ST8672 | VT | 7A/F | Unknown | 2004 | Illumina |
| A41543 | ERS012065 | ERR028402 | ERA020597 | SC11 | Blood | ST8672 | VT | 7A/F | Unknown | 2006 | Illumina |
| A36949 | ERS012130 | ERR028448 | ERA020597 | SC11 | Blood | ST8672 | VT | 7A/F | Female | 2005 | Illumina |
| D34296 | ERS012144 | ERR028463 | ERA020597 | SC11 | Blood | ST8672 | VT | 7A/F | Male | 2005 | Illumina |
| A29030 | ERS050332 | ERR068173 | ERA080989 | SC11 | Blood | ST8672 | VT | 7A/F | Female | 2004 | Illumina |
| A32849 | ERS050347 | ERR068188 | ERA080989 | SC11 | Blood | ST8672 | VT | 7A/F | Male | 2004 | Illumina |
| A42552 | ERS050374 | ERR068215 | ERA080989 | SC11 | Blood | ST10675 | VT | 7A/F | Unknown | 2006 | Illumina |
| A51189 | ERS050387 | ERR068228 | ERA080989 | SC11 | Blood | ST8672 | VT | 7A/F | Male | 2008 | Illumina |
| C10592 | ERS024519 | ERR033127 | ERA026370 | SC11 | CSF | ST8672 | VT | 7A/F | Male | 2004 | Illumina |
| B14208 | ERS008344 | ERR024522 | ERA015791 | SC11 | CSF | ST8672 | VT | 7A/F | Male | 2006 | Illumina |
| B18374 | ERS008348 | ERR024526 | ERA015791 | SC11 | CSF | ST8672 | VT | 7A/F | Male | 2008 | Illumina |
| B18408 | ERS050421 | ERR085972 | ERA087412 | SC11 | CSF | ST8672 | VT | 7A/F | Male | 2008 | Illumina |
| B19805 | ERS050428 | ERR085979 | ERA087412 | SC11 | CSF | ST11769 | VT | 7A/F | Female | 2009 | Illumina |
| C18075 | ERS050460 | ERR086011 | ERA087412 | SC11 | CSF | ST8672 | VT | 7A/F | Female | 2008 | Illumina |
| C20505 | ERS050471 | ERR086022 | ERA087412 | SC11 | CSF | ST8672 | VT | 7A/F | Male | 2009 | Illumina |
| BAN19Q | ERS050228 | ERR068070 | ERA080989 | SC11 | Carriage | ST8672 | VT | 7A/F | Male | 2011 | Illumina |
| D38588 | ERS006724 | ERR018024 | ERA012034 | SC12 | Blood | ST2678 | VT | 14 | Female | 2006 | Illumina |
| D25696 | ERS006736 | ERR018037 | ERA012034 | SC12 | Blood | ST63 | VT | 14 | Female | 2004 | Illumina |
| A42335 | ERS006794 | ERR018086 | ERA012034 | SC12 | Blood | ST5435 | VT | 3 | Unknown | 2006 | Illumina |
| A50302 | ERS006798 | ERR018101 | ERA012034 | SC12 | Blood | ST9171~ | NVT | 37 | Female | 2008 | Illumina |
| A30615 | ERS008319 | ERR024492 | ERA015791 | SC12 | Blood | ST10608 | NVT | 33A | Female | 2004 | Illumina |
| A41722 | ERS012066 | ERR028403 | ERA020597 | SC12 | Blood | ST63 | VT | 14 | Unknown | 2006 | Illumina |
| A28640 | ERS012092 | ERR028421 | ERA020597 | SC12 | Blood | ST63 | VT | 14 | Female | 2004 | Illumina |
| A37501 | ERS050367 | ERR068208 | ERA080989 | SC12 | Blood | ST63 | VT | 14 | Female | 2005 | Illumina |
| C17361 | ERS024522 | ERR033148 | ERA026370 | SC12 | CSF | ST63 | VT | 14 | Male | 2007 | Illumina |
| B9632 | ERS008350 | ERR024528 | ERA015791 | SC12 | CSF | ST10608 | NVT | 33A | Male | 2004 | Illumina |
| C9938 | ERS008358 | ERR024532 | ERA015791 | SC12 | CSF | ST63 | VT | 14 | Male | 2004 | Illumina |
| B12589 | ERS012136 | ERR028445 | ERA020597 | SC12 | CSF | ST63 | VT | 14 | Male | 2005 | Illumina |
| C18913 | ERS050465 | ERR086016 | ERA087412 | SC12 | CSF | ST63 | VT | 14 | Female | 2008 | Illumina |
| C18952 | ERS050467 | ERR086018 | ERA087412 | SC12 | CSF | ST63 | VT | 14 | Female | 2008 | Illumina |
| C21008 | ERS050477 | ERR086028 | ERA087412 | SC12 | CSF | ST63 | VT | 14 | Female | 2009 | Illumina |
| BAN152 | ERS050211 | ERR068053 | ERA080989 | SC12 | Carriage | ST5435 | VT | 3 | Female | 2011 | Illumina |
| BAN1C0 | ERS050243 | ERR068085 | ERA080989 | SC12 | Carriage | ST5435 | VT | 3 | Male | 2011 | Illumina |
| BAN15E | ERS050213 | ERR068055 | ERA080989 | SC12 | Carriage | ST63 | VT | 14 | Male | 2011 | Illumina |
| BAN1EP | ERS050254 | ERR068096 | ERA080989 | SC12 | Carriage | ST63 | VT | 14 | Male | 2011 | Illumina |
| BAN1GH | ERS050262 | ERR068104 | ERA080989 | SC12 | Carriage | ST63 | VT | 14 | Female | 2011 | Illumina |
| BAN1HC | ERS050265 | ERR068107 | ERA080989 | SC12 | Carriage | ST63 | VT | 14 | Male | 2011 | Illumina |
| BAN15R | ERS050215 | ERR068057 | ERA080989 | SC12 | Carriage | ST5435 | VT | 3 | Male | 2011 | Illumina |
| A29858 | ERS006790 | ERR018093 | ERA012034 | SC13 | Blood | ST9523 | VT | 18B/C | Female | 2004 | Illumina |
| D47510 | ERS024532 | ERR033152 | ERA026370 | SC13 | Blood | ST10868~ | NVT | 8 | Male | 2008 | Illumina |
| D28531 | ERS006818 | ERR023387 | ERA015706 | SC13 | Blood | ST9537~ | NVT | 18A | Unknown | 2004 | Illumina |
| A47266 | ERS003572 | ERR023980 | ERA015737 | SC13 | Blood | ST2213 | VT | 4 | Male | 2007 | Illumina |
| A52597 | ERS003582 | ERR023991 | ERA015737 | SC13 | Blood | ST10868 | NVT | 8 | Female | 2008 | Illumina |
| A43965 | ERS008332 | ERR024509 | ERA015791 | SC13 | Blood | ST2213 | VT | 4 | Unknown | 2006 | Illumina |
| D33733 | ERS008361 | ERR024535 | ERA015791 | SC13 | Blood | ST9523 | VT | 18B/C | Male | 2005 | Illumina |
| D46797 | ERS008364 | ERR024538 | ERA015791 | SC13 | Blood | ST9537~ | NVT | 18A | Male | 2008 | Illumina |
| A40807 | ERS012062 | ERR028399 | ERA020597 | SC13 | Blood | ST9523 | VT | 18B/C | Unknown | 2006 | Illumina |
| A33922 | ERS012119 | ERR028437 | ERA020597 | SC13 | Blood | ST2213 | VT | 4 | Female | 2005 | Illumina |
| A34562 | ERS012124 | ERR028442 | ERA020597 | SC13 | Blood | ST1871 | NVT | 9A | Male | 2005 | Illumina |
| A31265 | ERS050339 | ERR068180 | ERA080989 | SC13 | Blood | ST5396 | NVT | 35B | Male | 2004 | Illumina |
| A32279 | ERS050344 | ERR068185 | ERA080989 | SC13 | Blood | ST2213 | VT | 4 | Female | 2004 | Illumina |
| A36646 | ERS050358 | ERR068199 | ERA080989 | SC13 | Blood | ST2213 | VT | 4 | Female | 2005 | Illumina |
| A50227 | ERS050385 | ERR068226 | ERA080989 | SC13 | Blood | ST2213 | VT | 4 | Male | 2008 | Illumina |
| A51119 | ERS050386 | ERR068227 | ERA080989 | SC13 | Blood | ST9926 | VT | 6A | Unknown | 2008 | Illumina |
| C14560 | ERS006737 | ERR018035 | ERA012034 | SC13 | CSF | ST2213 | VT | 4 | Female | 2006 | Illumina |
| C10300 | ERS024518 | ERR033128 | ERA026370 | SC13 | CSF | ST1871 | NVT | 9A | Male | 2004 | Illumina |
| B18747 | ERS003579 | ERR023988 | ERA015737 | SC13 | CSF | ST1871 | NVT | 9A | Male | 2008 | Illumina |
| B9521 | ERS012108 | ERR026628 | ERA015971 | SC13 | CSF | ST1871 | NVT | 9A | Male | 2004 | Illumina |
| B14721 | ERS012071 | ERR028398 | ERA020597 | SC13 | CSF | ST5396 | NVT | 35B | Male | 2006 | Illumina |
| B15143 | ERS012074 | ERR028412 | ERA020597 | SC13 | CSF | ST10868~ | NVT | 8 | Female | 2006 | Illumina |
| B11670 | ERS050412 | ERR085963 | ERA087412 | SC13 | CSF | ST9926 | NVT | 17F | Male | 2005 | Illumina |
| B19554 | ERS050425 | ERR085976 | ERA087412 | SC13 | CSF | ST9523 | VT | 18B/C | Male | 2009 | Illumina |
| B19728 | ERS050427 | ERR085978 | ERA087412 | SC13 | CSF | ST5396 | NVT | 35B | Female | 2009 | Illumina |
| C12675 | ERS050452 | ERR086003 | ERA087412 | SC13 | CSF | ST9523 | VT | 18B/C | Male | 2005 | Illumina |
| C17711 | ERS050458 | ERR086009 | ERA087412 | SC13 | CSF | ST7053 | NVT | 7C | Female | 2008 | Illumina |
| C21085 | ERS050479 | ERR086030 | ERA087412 | SC13 | CSF | ST9537~ | NVT | 18A | Male | 2009 | Illumina |
| C23125 | ERS050482 | ERR086033 | ERA087412 | SC13 | CSF | ST9523 | VT | 18B/C | Female | 2010 | Illumina |
| BAN19R | ERS050229 | ERR068071 | ERA080989 | SC13 | Carriage | ST2213 | VT | 4 | Male | 2011 | Illumina |
| BAN1AS | ERS050233 | ERR068075 | ERA080989 | SC13 | Carriage | ST5396 | NVT | 35B | Male | 2011 | Illumina |
| BAN1BC | ERS050238 | ERR068080 | ERA080989 | SC13 | Carriage | ST10868 | NVT | 8 | Male | 2010 | Illumina |
| BAN1E7 | ERS050251 | ERR068093 | ERA080989 | SC13 | Carriage | ST7105 | NVT | 7C | Male | 2011 | Illumina |
| BAN1ME | ERS050279 | ERR068121 | ERA080989 | SC13 | Carriage | ST7053 | NVT | 7C | Female | 2011 | Illumina |
| BAN1RK | ERS050295 | ERR068137 | ERA080989 | SC13 | Carriage | ST7053 | VT | 19F | Female | 2011 | Illumina |
| BAN1SX | ERS050301 | ERR068143 | ERA080989 | SC13 | Carriage | ST10587 | NVT | 7C | Male | 2011 | Illumina |
| BAN1VE | ERS050312 | ERR068153 | ERA080989 | SC13 | Carriage | ST3265 | NVT | NT | Female | 2011 | Illumina |
| D41088 | ERS006750 | ERR018049 | ERA012034 | SC14 | Blood | ST4914~ | NVT | 9L | Male | 2007 | Illumina |
| D47322 | ERS006756 | ERR018056 | ERA012034 | SC14 | Blood | ST347 | VT | 19F | Female | 2008 | Illumina |
| D46184 | ERS006757 | ERR018057 | ERA012034 | SC14 | Blood | ST9575~ | VT | 6A | Female | 2008 | Illumina |
| A34964 | ERS006775 | ERR018077 | ERA012034 | SC14 | Blood | ST9532 | VT | 6A | Male | 2005 | Illumina |
| A37855 | ERS006786 | ERR018088 | ERA012034 | SC14 | Blood | ST1004~ | VT | 4 | Male | 2005 | Illumina |
| A28899 | ERS006791 | ERR018094 | ERA012034 | SC14 | Blood | ST5266 | NVT | 22A/F | Male | 2004 | Illumina |
| A25958 | ERS006800 | ERR018104 | ERA012034 | SC14 | Blood | ST2927 | VT | 18B/C | Unknown | 2003 | Illumina |
| A43382 | ERS006805 | ERR018109 | ERA012034 | SC14 | Blood | ST9445 | NVT | 45 | Unknown | 2006 | Illumina |
| D24904 | ERS024515 | ERR033131 | ERA026370 | SC14 | Blood | ST5080 | NVT | 23A | Male | 2004 | Illumina |
| D30974 | ERS024513 | ERR033133 | ERA026370 | SC14 | Blood | ST10874 | NVT | 9A | Unknown | 2005 | Illumina |
| D32289 | ERS026040 | ERR033137 | ERA026370 | SC14 | Blood | ST5393~ | VT | 19F | Unknown | 2005 | Illumina |
| D36355 | ERS026049 | ERR033139 | ERA026370 | SC14 | Blood | ST2285 | VT | 6A | Male | 2006 | Illumina |
| D32344 | ERS026043 | ERR033145 | ERA026370 | SC14 | Blood | ST10595 | NVT | 13 | Unknown | 2005 | Illumina |
| D47292 | ERS024530 | ERR033154 | ERA026370 | SC14 | Blood | ST8267 | VT | 6A | Male | 2008 | Illumina |
| A51396 | ERS003568 | ERR023975 | ERA015737 | SC14 | Blood | ST5847~ | NVT | 31 | Male | 2008 | Illumina |
| A47814 | ERS003571 | ERR023979 | ERA015737 | SC14 | Blood | ST2650 | NVT | 25A/F | Male | 2007 | Illumina |
| A51546 | ERS003587 | ERR023987 | ERA015737 | SC14 | Blood | ST10568~ | NVT | 11D | Male | 2008 | Illumina |
| A51397 | ERS003581 | ERR023990 | ERA015737 | SC14 | Blood | ST5847~ | NVT | 31 | Female | 2008 | Illumina |
| A36530 | ERS008325 | ERR024501 | ERA015791 | SC14 | Blood | ST7063 | VT | 6A | Male | 2005 | Illumina |
| A47073 | ERS008333 | ERR024510 | ERA015791 | SC14 | Blood | ST5412 | VT | 6A | Female | 2007 | Illumina |
| D31827 | ERS008359 | ERR024533 | ERA015791 | SC14 | Blood | ST9546~ | NVT | 34 | Unknown | 2005 | Illumina |
| A29167 | ERS012096 | ERR026615 | ERA015971 | SC14 | Blood | ST5902 | NVT | 9A | Male | 2004 | Illumina |
| A30277 | ERS012098 | ERR026617 | ERA015971 | SC14 | Blood | ST5902 | NVT | 9A | Female | 2004 | Illumina |
| A33054 | ERS012114 | ERR026624 | ERA015971 | SC14 | Blood | ST3548 | NVT | 31 | Male | 2005 | Illumina |
| D28166 | ERS012113 | ERR026633 | ERA015971 | SC14 | Blood | ST102 | VT | 18B/C | Female | 2004 | Illumina |
| A41848 | ERS012067 | ERR028404 | ERA020597 | SC14 | Blood | ST10568~ | VT | 19F | Unknown | 2006 | Illumina |
| D38834 | ERS012080 | ERR028418 | ERA020597 | SC14 | Blood | ST2053 | NVT | 13 | Female | 2006 | Illumina |
| A34594 | ERS012126 | ERR028434 | ERA020597 | SC14 | Blood | ST9471 | NVT | 9L | Unknown | 2005 | Illumina |
| A33813 | ERS012117 | ERR028435 | ERA020597 | SC14 | Blood | ST5266~ | NVT | 9A | Female | 2005 | Illumina |
| A34292 | ERS012123 | ERR028441 | ERA020597 | SC14 | Blood | ST5778 | NVT | 9A | Female | 2005 | Illumina |
| A39048 | ERS012146 | ERR028456 | ERA020597 | SC14 | Blood | ST2053 | NVT | 13 | Unknown | 2006 | Illumina |
| D33275 | ERS012143 | ERR028462 | ERA020597 | SC14 | Blood | ST9533 | VT | 6A | Male | 2005 | Illumina |
| D36051 | ERS012145 | ERR028464 | ERA020597 | SC14 | Blood | ST9532 | VT | 6A | Female | 2005 | Illumina |
| A29265 | ERS050333 | ERR068174 | ERA080989 | SC14 | Blood | ST5077~ | NVT | 24F | Male | 2004 | Illumina |
| A31122 | ERS050336 | ERR068177 | ERA080989 | SC14 | Blood | ST9533 | VT | 6A | Male | 2004 | Illumina |
| A32497 | ERS050345 | ERR068186 | ERA080989 | SC14 | Blood | ST5077 | NVT | 24F | Male | 2004 | Illumina |
| A36611 | ERS050357 | ERR068198 | ERA080989 | SC14 | Blood | ST5847~ | NVT | 31 | Female | 2005 | Illumina |
| A41112 | ERS050371 | ERR068212 | ERA080989 | SC14 | Blood | ST10583 | NVT | 12B | Unknown | 2006 | Illumina |
| A42501 | ERS050373 | ERR068214 | ERA080989 | SC14 | Blood | ST2650 | NVT | 25A/F | Unknown | 2006 | Illumina |
| A49649 | ERS050384 | ERR068225 | ERA080989 | SC14 | Blood | ST2650 | NVT | 25A/F | Male | 2008 | Illumina |
| A51905 | ERS050389 | ERR068230 | ERA080989 | SC14 | Blood | ST6030 | NVT | 16F | Female | 2008 | Illumina |
| A51983 | ERS050391 | ERR068232 | ERA080989 | SC14 | Blood | ST3207~ | VT | 6A | Male | 2008 | Illumina |
| A53861 | ERS050398 | ERR068239 | ERA080989 | SC14 | Blood | ST9546 | NVT | 22A/F | Male | 2009 | Illumina |
| A54575 | ERS050401 | ERR068242 | ERA080989 | SC14 | Blood | ST347 | VT | 19F | Female | 2009 | Illumina |
| D38708 | ERS050636 | ERR085857 | ERA087412 | SC14 | Blood | ST347 | VT | 19F | Male | 2006 | Illumina |
| D39094 | ERS050637 | ERR085858 | ERA087412 | SC14 | Blood | ST2053 | NVT | 13 | Male | 2006 | Illumina |
| A58785 | ERS050406 | ERR085957 | ERA087412 | SC14 | Blood | ST8267~ | VT | 6A | Male | 2010 | Illumina |
| D27950 | ERS050495 | ERR086046 | ERA087412 | SC14 | Blood | ST10761~ | NVT | 15A | Female | 2004 | Illumina |
| D38557 | ERS050497 | ERR086048 | ERA087412 | SC14 | Blood | ST10583 | NVT | 12B | Male | 2006 | Illumina |
| C14376 | ERS006732 | ERR018030 | ERA012034 | SC14 | CSF | ST9572~ | NVT | 15B/C | Male | 2006 | Illumina |
| C14132 | ERS006735 | ERR018033 | ERA012034 | SC14 | CSF | ST9936 | NVT | 15B/C | Female | 2006 | Illumina |
| C18673 | ERS006739 | ERR018038 | ERA012034 | SC14 | CSF | ST2285 | VT | 6A | Female | 2008 | Illumina |
| C16847 | ERS006742 | ERR018041 | ERA012034 | SC14 | CSF | ST347 | VT | 19F | Male | 2007 | Illumina |
| B18037 | ERS006801 | ERR018105 | ERA012034 | SC14 | CSF | ST10583 | NVT | 12B | Male | 2008 | Illumina |
| C10281 | ERS024520 | ERR033126 | ERA026370 | SC14 | CSF | ST102 | VT | 18B/C | Male | 2004 | Illumina |
| C13260 | ERS026048 | ERR033140 | ERA026370 | SC14 | CSF | ST5266~ | VT | 18B/C | Male | 2005 | Illumina |
| B11188 | ERS003554 | ERR023971 | ERA015737 | SC14 | CSF | ST9445 | NVT | 45 | Female | 2005 | Illumina |
| B11811 | ERS003556 | ERR023973 | ERA015737 | SC14 | CSF | ST9445 | NVT | 45 | Male | 2005 | Illumina |
| B15467 | ERS003574 | ERR023982 | ERA015737 | SC14 | CSF | ST9445 | NVT | 45 | Male | 2007 | Illumina |
| B18487 | ERS003585 | ERR023994 | ERA015737 | SC14 | CSF | ST2053 | NVT | 13 | Female | 2008 | Illumina |
| B12226 | ERS008340 | ERR024506 | ERA015791 | SC14 | CSF | ST7063 | VT | 6A | Female | 2005 | Illumina |
| B18762 | ERS008349 | ERR024527 | ERA015791 | SC14 | CSF | ST9546~ | NVT | 34 | Male | 2008 | Illumina |
| B8923 | ERS012104 | ERR026613 | ERA015971 | SC14 | CSF | ST5604 | NVT | 25A/F | Male | 2004 | Illumina |
| B10027 | ERS012100 | ERR026619 | ERA015971 | SC14 | CSF | ST5902 | NVT | 9A | Male | 2004 | Illumina |
| B10622 | ERS012102 | ERR026621 | ERA015971 | SC14 | CSF | ST2773~ | NVT | 15B/C | Female | 2004 | Illumina |
| B9734 | ERS012110 | ERR026630 | ERA015971 | SC14 | CSF | ST10633~ | NVT | 11D | Female | 2004 | Illumina |
| B17790 | ERS012089 | ERR028428 | ERA020597 | SC14 | CSF | ST3544 | VT | 7A/F | Female | 2008 | Illumina |
| B11223 | ERS012132 | ERR028450 | ERA020597 | SC14 | CSF | ST10583 | NVT | 12B | Female | 2005 | Illumina |
| B11855 | ERS012135 | ERR028453 | ERA020597 | SC14 | CSF | ST5778 | NVT | 9A | Female | 2005 | Illumina |
| B12658 | ERS012137 | ERR028455 | ERA020597 | SC14 | CSF | ST10568~ | VT | 19F | Male | 2005 | Illumina |
| B18210 | ERS050419 | ERR085970 | ERA087412 | SC14 | CSF | ST9445 | NVT | 45 | Female | 2008 | Illumina |
| B19190 | ERS050424 | ERR085975 | ERA087412 | SC14 | CSF | ST9484~ | VT | 18B/C | Female | 2009 | Illumina |
| B19944 | ERS050429 | ERR085980 | ERA087412 | SC14 | CSF | ST5079 | NVT | 24A | Female | 2009 | Illumina |
| B20065 | ERS050430 | ERR085981 | ERA087412 | SC14 | CSF | ST2234 | NVT | 8 | Male | 2009 | Illumina |
| B20084 | ERS050431 | ERR085982 | ERA087412 | SC14 | CSF | ST9546 | NVT | 22A/F | Female | 2009 | Illumina |
| B20631 | ERS050434 | ERR085985 | ERA087412 | SC14 | CSF | ST2650 | NVT | 25A/F | Female | 2009 | Illumina |
| B20776 | ERS050435 | ERR085986 | ERA087412 | SC14 | CSF | ST4221~ | NVT | 9A | Male | 2009 | Illumina |
| B21503 | ERS050436 | ERR085987 | ERA087412 | SC14 | CSF | ST2053 | NVT | 13 | Male | 2010 | Illumina |
| B21600 | ERS050438 | ERR085989 | ERA087412 | SC14 | CSF | ST7653~ | VT | 6D | Male | 2010 | Illumina |
| B21950 | ERS050440 | ERR085991 | ERA087412 | SC14 | CSF | ST6030 | NVT | 16F | Female | 2010 | Illumina |
| B21982 | ERS050442 | ERR085993 | ERA087412 | SC14 | CSF | ST9936 | NVT | 15B/C | Male | 2010 | Illumina |
| C12480 | ERS050451 | ERR086002 | ERA087412 | SC14 | CSF | ST9533 | VT | 6A | Male | 2005 | Illumina |
| C12879 | ERS050454 | ERR086005 | ERA087412 | SC14 | CSF | ST5077 | NVT | 24F | Male | 2005 | Illumina |
| C14257 | ERS050455 | ERR086006 | ERA087412 | SC14 | CSF | ST9471 | NVT | 9L | Female | 2006 | Illumina |
| C14277 | ERS050456 | ERR086007 | ERA087412 | SC14 | CSF | ST2053 | NVT | 13 | Female | 2006 | Illumina |
| C14971 | ERS050457 | ERR086008 | ERA087412 | SC14 | CSF | ST10583 | NVT | 12B | Female | 2006 | Illumina |
| C18113 | ERS050461 | ERR086012 | ERA087412 | SC14 | CSF | ST9572 | NVT | 15B/C | Female | 2008 | Illumina |
| C19000 | ERS050468 | ERR086019 | ERA087412 | SC14 | CSF | ST2927 | VT | 18B/C | Male | 2008 | Illumina |
| C20393 | ERS050470 | ERR086021 | ERA087412 | SC14 | CSF | ST2773~ | NVT | 15B/C | Male | 2009 | Illumina |
| C20650 | ERS050472 | ERR086023 | ERA087412 | SC14 | CSF | ST5778~ | NVT | 9A | Male | 2009 | Illumina |
| C20742 | ERS050473 | ERR086024 | ERA087412 | SC14 | CSF | ST5266 | VT | 18B/C | Male | 2009 | Illumina |
| C20932 | ERS050476 | ERR086027 | ERA087412 | SC14 | CSF | ST9473 | NVT | 9A | Male | 2009 | Illumina |
| C23171 | ERS050483 | ERR086034 | ERA087412 | SC14 | CSF | ST9903 | VT | 18B/C | Female | 2010 | Illumina |
| BAN17V | ERS050219 | ERR068061 | ERA080989 | SC14 | Carriage | ST347 | VT | 19F | Male | 2011 | Illumina |
| BAN18M | ERS050222 | ERR068064 | ERA080989 | SC14 | Carriage | ST11774 | NVT | 21 | Male | 2011 | Illumina |
| BAN19G | ERS050226 | ERR068068 | ERA080989 | SC14 | Carriage | ST5266~ | VT | 6A | Female | 2011 | Illumina |
| BAN1AL | ERS050231 | ERR068073 | ERA080989 | SC14 | Carriage | ST9476 | NVT | 2 | Female | 2011 | Illumina |
| BAN1AR | ERS050232 | ERR068074 | ERA080989 | SC14 | Carriage | ST2285 | VT | 6A | Male | 2011 | Illumina |
| BAN1AU | ERS050235 | ERR068077 | ERA080989 | SC14 | Carriage | ST9546 | NVT | 34 | Female | 2011 | Illumina |
| BAN1BV | ERS050242 | ERR068084 | ERA080989 | SC14 | Carriage | ST6030 | NVT | 16F | Female | 2011 | Illumina |
| BAN1C7 | ERS050245 | ERR068087 | ERA080989 | SC14 | Carriage | ST6030 | NVT | 16F | Male | 2011 | Illumina |
| BAN1CR | ERS050246 | ERR068088 | ERA080989 | SC14 | Carriage | ST10568 | NVT | 11D | Female | 2011 | Illumina |
| BAN1D8 | ERS050247 | ERR068089 | ERA080989 | SC14 | Carriage | ST9491 | VT | 23F | Female | 2011 | Illumina |
| BAN1DE | ERS050248 | ERR068090 | ERA080989 | SC14 | Carriage | ST3214 | NVT | 42 | Male | 2011 | Illumina |
| BAN15N | ERS050214 | ERR068056 | ERA080989 | SC14 | Carriage | ST10633 | NVT | 11D | Female | 2011 | Illumina |
| BAN1GB | ERS050259 | ERR068101 | ERA080989 | SC14 | Carriage | ST10674 | NVT | 23B | Male | 2011 | Illumina |
| BAN1GE | ERS050260 | ERR068102 | ERA080989 | SC14 | Carriage | ST5483 | NVT | 29 | Male | 2011 | Illumina |
| BAN1HX | ERS050267 | ERR068109 | ERA080989 | SC14 | Carriage | ST4914~ | NVT | 9L | Male | 2011 | Illumina |
| BAN1I6 | ERS050268 | ERR068110 | ERA080989 | SC14 | Carriage | ST2074~ | NVT | 9L | Male | 2011 | Illumina |
| BAN1I8 | ERS050269 | ERR068111 | ERA080989 | SC14 | Carriage | ST11709 | VT | 6A | Male | 2011 | Illumina |
| BAN1KA | ERS050277 | ERR068119 | ERA080989 | SC14 | Carriage | ST3214 | NVT | 42 | Male | 2011 | Illumina |
| BAN1KT | ERS050278 | ERR068120 | ERA080989 | SC14 | Carriage | ST3214 | NVT | 35C | Female | 2011 | Illumina |
| BAN15S | ERS050216 | ERR068058 | ERA080989 | SC14 | Carriage | ST5080 | NVT | 23A | Male | 2011 | Illumina |
| BAN1PY | ERS050282 | ERR068124 | ERA080989 | SC14 | Carriage | ST6103 | NVT | 38 | Male | 2011 | Illumina |
| BAN1Q3 | ERS050283 | ERR068125 | ERA080989 | SC14 | Carriage | ST847 | VT | 19A | Male | 2011 | Illumina |
| BAN1QF | ERS050286 | ERR068128 | ERA080989 | SC14 | Carriage | ST9471 | NVT | 9L | Female | 2011 | Illumina |
| BAN1QR | ERS050288 | ERR068130 | ERA080989 | SC14 | Carriage | ST9915 | VT | 6A | Male | 2011 | Illumina |
| BAN1QZ | ERS050290 | ERR068132 | ERA080989 | SC14 | Carriage | ST7063 | VT | 6A | Male | 2011 | Illumina |
| BAN1R9 | ERS050291 | ERR068133 | ERA080989 | SC14 | Carriage | ST2795 | NVT | 35A | Female | 2011 | Illumina |
| BAN1RB | ERS050293 | ERR068135 | ERA080989 | SC14 | Carriage | ST9532 | VT | 6A | Female | 2011 | Illumina |
| BAN1RH | ERS050294 | ERR068136 | ERA080989 | SC14 | Carriage | ST6030 | NVT | 16F | Male | 2011 | Illumina |
| BAN1S3 | ERS050296 | ERR068138 | ERA080989 | SC14 | Carriage | ST10761 | NVT | 15A | Male | 2011 | Illumina |
| BAN1SV | ERS050300 | ERR068142 | ERA080989 | SC14 | Carriage | ST9572 | NVT | 15B/C | Female | 2011 | Illumina |
| BAN1TT | ERS050304 | ERR068146 | ERA080989 | SC14 | Carriage | ST10660 | VT | 6A | Male | 2011 | Illumina |
| BAN1TU | ERS050305 | ERR068147 | ERA080989 | SC14 | Carriage | ST5768~ | NVT | 15A | Female | 2011 | Illumina |
| BAN22F | ERS050316 | ERR068157 | ERA080989 | SC14 | Carriage | ST2053 | NVT | 13 | Female | 2011 | Illumina |
| BAN23T | ERS050317 | ERR068158 | ERA080989 | SC14 | Carriage | ST1264 | NVT | 15B/C | Female | 2011 | Illumina |
| BAN26E | ERS050322 | ERR068163 | ERA080989 | SC14 | Carriage | ST2234 | NVT | 8 | Male | 2011 | Illumina |
| BAN26N | ERS050323 | ERR068164 | ERA080989 | SC14 | Carriage | ST6030 | NVT | 16F | Male | 2011 | Illumina |
| BAN223 | ERS050315 | ERR068156 | ERA080989 | SC14 | Carriage | ST9546 | NVT | 34 | Female | 2011 | Illumina |

Vaccine Status: Vaccine Type (VT), NVT (Non-Vaccine Type). STs that were not available in the pneumococcal MLST database are designated with their closest ST (~ at the end).
