# Supplementary material for: Recombination in Streptococcus pneumoniae Lineages Increase with Carriage Duration and Size of the Polysaccharide Capsule
Source: mBio. 2016 Sep 27;7(5):e01053-16. doi: 10.1128/mBio.01053-16 (PMC5040112; doi:10.1128/mBio.01053-16)
Supplement: Table S2 — Reference pneumococcal whole-genome sequences used in this study. [file mbo005163006st2.docx]

**Table S2:** Reference *S. pneumoniae* genomes used in the study

| **Accession ID** | **Serotype** | **Common ID** |
| --- | --- | --- |
| NC_014498 | 6B | 670-6B |
| NC_003028 | 4 | TIGR4 |
| NC_010380 | 19A | Hungary19A |
| NC_017592 | 3 | OXC141 |
| NC_011900 | 23F | ATCC700669 |
| CACE00000000 | 1 | SPN1041 |
| NC_003028 | Other serotypes with no fully finished reference genomes | TIGR4 |
