# Supplementary material for: Recombination in Streptococcus pneumoniae Lineages Increase with Carriage Duration and Size of the Polysaccharide Capsule
Source: mBio. 2016 Sep 27;7(5):e01053-16. doi: 10.1128/mBio.01053-16 (PMC5040112; doi:10.1128/mBio.01053-16)
Supplement: Table S3 — Summarized estimates from the univariate and multivariable regression. Only serotypes with all the different independent variables present, i.e., capsule size, carriage duration, invasive potential, and carriage prevalence were used. [file mbo005163006st3.docx]

| Response Variables | Predictor Variable | Univariate | | |  | Multivariable | | |
| --- | --- | --- | --- | --- | --- | --- | --- | --- |
|  |  | **Estimate** | **Standard Error** | **P-value** |  | **Estimate** | **Standard Error** | **P-value** |
| Recombination  Rate (μ*_r/m_*) | Capsule Size | 0.0147 | 0.0201 | 0.4888 |  | -0.0185 | 0.0242 | 0.4869 |
|  | Carriage Duration | 0.3722 | 0.1006 | 0.0076* |  | 0.2149 | 0.3301 | 0.5504 |
|  | Invasive Potential | -1.0604 | 0.4232 | 0.0407* |  | -0.8847 | 0.7927 | 0.3269 |
|  | Carriage Prevalence | 1.0765 | 0.3395 | 0.0157* |  | 0.1081 | 0.9468 | 0.9146 |
| Recombination  Frequency (μ_re_) | Capsule Size | 0.0251 | 0.0085 | 0.0209* |  | 0.0057 | 0.0081 | 0.5163 |
|  | Carriage Duration | 0.2134 | 0.0681 | 0.0165* |  | 0.2595 | 0.1101 | 0.0780 |
|  | Invasive Potential | -0.8342 | 0.1647 | 0.0015* |  | -0.4127 | 0.2645 | 0.1937 |
|  | Carriage Prevalence | 0.4453 | 0.2761 | 0.1508 |  | -0.4967 | 0.3159 | 0.1910 |

The asterisk (*) in the p-value columns indicates significant terms.
